# Transcriptomic responses to environmental temperature by turtles with temperature-dependent and genotypic sex determination assessed by RNAseq inform the genetic architecture of embryonic gonadal development

**DOI:** 10.1371/journal.pone.0172044

**Published:** 2017-03-15

**Authors:** Srihari Radhakrishnan, Robert Literman, Jennifer Neuwald, Andrew Severin, Nicole Valenzuela

**Affiliations:** 1 Bioinformatics and Computational Biology Program, Iowa State University, Ames, IA, United States of America; 2 Department of Ecology, Evolution and Organismal Biology, Iowa State University, Ames, IA, United States of America; 3 Ecology and Evolutionary Biology Program, Iowa State University, Ames, IA, United States of America; 4 Genome Informatics Facility, Iowa State University, Ames, IA, United States of America; Leibniz Institute on aging - Fritz Lipmann Institute (FLI), GERMANY

## Abstract

Vertebrate sexual fate is decided primarily by the individual’s genotype (GSD), by the environmental temperature during development (TSD), or both. Turtles exhibit TSD and GSD, making them ideal to study the evolution of sex determination. Here we analyze temperature-specific gonadal transcriptomes (RNA-sequencing validated by qPCR) of painted turtles (*Chrysemys picta* TSD) before and during the thermosensitive period, and at equivalent stages in soft-shell turtles (*Apalone spinifera—*GSD), to test whether TSD’s and GSD’s transcriptional circuitry is identical but deployed differently between mechanisms. Our data show that most elements of the mammalian urogenital network are active during turtle gonadogenesis, but their transcription is generally more thermoresponsive in TSD than GSD, and concordant with their sex-specific function in mammals [e.g., upregulation of *Amh*, *Ar*, *Esr1*, *Fog2*, *Gata4*, *Igf1r*, *Insr*, and *Lhx9* at male-producing temperature, and of *β-catenin*, *Foxl2*, *Aromatase* (*Cyp19a1*), *Fst*, *Nf-kb*, *Crabp2* at female-producing temperature in *Chrysemys*]. Notably, antagonistic elements in gonadogenesis (e.g., *β-catenin* and *Insr*) were thermosensitive only in TSD early-embryos. *Cirbp* showed warm-temperature upregulation in both turtles disputing its purported key TSD role. Genes that may convert thermal inputs into sex-specific development (e.g., signaling and hormonal pathways, RNA-binding and heat-shock) were differentially regulated. *Jak-Stat*, *Nf-κB*, *retinoic-acid*, *Wnt*, and *Mapk-*signaling (not *Akt* and *Ras*-signaling) potentially mediate TSD thermosensitivity. Numerous species-specific ncRNAs (including *Xist*) were differentially-expressed, mostly upregulated at colder temperatures, as were unannotated loci that constitute novel TSD candidates. *Cirbp* showed warm-temperature upregulation in both turtles. Consistent transcription between turtles and alligator revealed putatively-critical reptilian TSD elements for male (*Sf1*, *Amh*, *Amhr2*) and female (*Crabp2* and *Hspb1*) gonadogenesis. In conclusion, while preliminary, our data helps illuminate the regulation and evolution of vertebrate sex determination, and contribute genomic resources to guide further research into this fundamental biological process.

## Introduction

Organisms vary wildly in how they determine sex [1]. Vertebrate sex-determining mechanisms range between genotypic sex determination (GSD) and environmental sex determination (ESD) [2, 3]. The most common ESD mechanism in vertebrates is temperature-dependent sex determination (TSD). The commitment of the bipotential gonad to differentiate into testes or ovaries is triggered by the genotype in GSD, and by temperatures experienced during the thermosensitive period of embryonic or larval development in TSD [4–6]. All studied mammals, birds and amphibians exhibit GSD, while TSD is found in some species of fish and lizards, tuatara, all crocodilians, and most turtles [2, 7]. GSD represents systems of extreme developmental canalization while TSD is a textbook example of phenotypic plasticity. Intermediate mechanisms also exist where the genotype is overridden by temperature [2, 8], even in the presence of sex chromosomes [9, 10]. TSD is ancestral to turtles, reptiles, and perhaps even amniotes [11–14] such that the TSD of turtles and crocodilians may be considered homologous traits [11, 14]. This diversity has defied scientific explanation [1], impeding a full understanding of sex ratio evolution and consequently of population dynamics and diversification both in the past and in the face of contemporary environmental change [15–18]. This gap is due partly to our incomplete understanding of the molecular basis of GSD and TSD. For instance, the key genetic elements that mediate the effect of environmental temperature in TSD systems remain elusive. Unlike GSD models such as mammals [19–22] and chicken [23] whose gonadal developmental pathways are well understood (albeit not fully), our knowledge for GSD reptiles and TSD species is incipient, and prevents deciphering the genetic architecture of vertebrate sex determination and its evolution.

The first reptilian gonadal transcriptomes were recently characterized in alligator (TSD) [24], and slider turtles (TSD)[25], whereas previous studies on GSD and TSD turtles had used exclusively quantitative PCR and in situ hybridization targeting a number of genes underlying sexual development (full gene names are found in Table 1), including *Wt1* [26, 27], *Sf1* [28, 29], *Dax1* [30, 31], *Sox9* [30, 32–34], *Aromatase* [35, 36], *Dmrt1* [30, 34, 37], *Estrogen receptor* [38, 39], *Rspo1* [33, 40], among others (S1 Table). While these earlier turtle studies provided only fragmentary information [30, 33, 41, 42], their use of sensitive qPCR permitted the detection of differential gene expression that can pass undetected in transcriptomic analyses [43, 44], and these efforts uncovered substantial evolution of transcriptional patterns for some elements across vertebrates. However, the extent of evolution of the composition and expression of this gene regulatory network remains unclear. Thus, deciphering the composition, environmental sensitivity, and evolution of the gonadal gene network in additional TSD turtles and how they compare to GSD turtles is overdue.

**Table 1:** Full names of the genes discussed in the text.

Gene symbol	Gene name
*Amh*	Anti-mullerian hormone
*Apc*	Adenomatous Polyposis Coli
*AR*	Androgen receptor
*Bcr*	breakpoint cluster region protein
*Calr*	Calreticulin
*Casp1*	Caspase 1
*Cbx2*	Chromobox protein homolog 2
*Ck1*	Casein kinase 1
*Cyp19a*	Aromatase
*Crabp2*	Cellular Retinoic Acid Binding Protein 2
*Ctnnb1*	Beta catenin1
*Dax1*	dosage-sensitive sex reversal, adrenal hypoplasia critical region, on chromosome X, gene 1
*Dcn*	Decorin
*Dmrt1*	Doublesex and mab-3 related transcription factor 1
*Dmrt2*	Doublesex and mab-3 related transcription factor 2
*Ep300*	E1A Binding Protein P300
*Emx2*	Empty Spiracles Homeobox 2
*Esr2*	Estrogen receptor 2
*Fgf9*	Fibroblast growth factor 9
*Fgfr2*	Fibroblast growth factor receptor 2
*Fhl2*	Four And A Half LIM Domains 2
*Fog2*	Friend of Gata Protein 2
*Foxl2*	Forkhead box L2
*Fst*	Follistatin
*Gata2*	Gata binding protein 2
*Git2*	G Protein-Coupled Receptor Kinase Interacting ArfGAP 2
*Gsk3*	Glycogen Synthase Kinase 3
*Hspx*	Heat-shock protein x
*Igf1r*	Insulin like growth factor 1 receptor
*Insr*	Insulin receptor
*Kdm3a*	(Lysine (K)-Specific Demethylase 3A
*Lhx1*	Lim homoeobox 1
*Mapk*	Mitogen activated protein kinase
*Nfk-B*	nuclear factor kappa-light-chain-enhancer of activated B cells
*Ptch1*	Patched1
*Rps6*	Ribosomal protein s6
*Rspo1*	R-spondin1
*Serpinh1*	serpin peptidase inhibitor, clade H (heat shock protein 47), member 1)
*Six1*	SIX Homeobox 1
*Six4*	SIX Homeobox 4
*Sf1*	Steroidogenic factor 1
*Sox9*	SRY (sex determining region Y)-box 9
*Srd5a2*	Steroid-5-Alpha-Reductase, Alpha Polypeptide 2
*Tcf21*	transcription factor 21
*Vegf*	Vascular endothelial growth factor
*Wnt4*	Wingless-Type MMTV Integration Site Family, Member 4
*Wt1*	Wilms tumor protein 1

Here, we use a comparative approach to test for transcriptional responses to environmental temperature (or lack thereof) at several stages of embryonic development in two turtle species, the painted turtle *Chrysemys picta* (TSD) and the soft-shell turtle *Apalone spinifera* (GSD), hereafter denoted as *Chrysemys* and *Apalone* respectively. *Chrysemys* is a TSD turtle lacking sex chromosomes [45] whose thermal ecology has been studied extensively [46–49], and constitutes an emerging model for ecology, evolution, and human health [50] with increasing genomic resources available [51–53]. *Apalone* is a GSD turtle with ZZ/ZW sex chromosomes [54] whose sex ratios are unaffected by the incubation temperature [55], and thus, it serves as a negative control for TSD responses.

Our RNAseq approach provides the first glimpse of the full transcriptional network in closely related reptiles with contrasting sex-determining mechanisms (GSD and TSD), and complements recent contrasts between turtles and model mammalian developing gonads [25], as well as enabling a comparison with alligator (another TSD reptile) [24]. With these data we carry out an initial test of several hypotheses to help elucidate whether the transcriptional building blocks in TSD and GSD systems are identical, and whether differential deployment of common elements distinguishes these systems. First, we examine whether the vertebrate sex determination/differentiation gene regulatory network known from model mammals and birds [3, 42, 56] is present and active in painted turtles. Second, we test whether the conversion of thermal inputs into TSD sex-specific development in painted turtles might involve the epigenetic machinery [57], hormonal pathways [58], or general sensing responses [59], by examining the transcriptional response to incubation temperature of heat-shock genes, transient receptor potential genes, germ-line and histone-related genes, as well as androgen- and estrogen related genes. Third, we test for differences in the transcriptional response to temperature between TSD and GSD turtles to uncover changes that may set these systems apart. Additionally, we identify novel candidate genes in painted turtles undescribed in mice and compare this information to that of other TSD reptiles [24, 25] to test whether they represent potentially unique reptilian regulators. Importantly, our transcriptomic time series of developing gonads at high and low temperatures in TSD and GSD turtles across developmental stages before, during and after the activation of the thermosensitive period (TSP) constitutes a critically needed resource to facilitate more extensive research to illuminate the proximate ecological regulation and evolution of vertebrate sex determination under various thermal regimes [16]. We note that for each species a single transcriptome was obtained from pooled embryos at each developmental stage at each temperature, such that our results are preliminary, and our conclusions represent critical working hypotheses to foster further research in this fascinating field.

## Materials and methods

### Sample collection

Total RNA was extracted using RNeasy Kits (Qiagen) [31] from *Chrysemys* (TSD) and *Apalone* (GSD). These species were chosen because their sex-determining mechanisms are well characterized [45, 46, 54, 60], and their abundance permits extensive sampling [50]. Embryos were collected at stages 9, 12, 15, 19 and 22 from eggs obtained from a turtle farm and incubated at 26°C and 31°C which are male- (MPT) and female-producing (FPT), respectively, in *Chrysemys*. Identical incubation conditions were used for *Apalone*, and these temperatures fall within the optimal thermal range for this species [55]. All procedures were approved by the IACUC committee of Iowa State University. Egg incubation followed previous description [42, 61], using boxes containing moistened sand. *Apalone* embryos were collected at the same stages as *Chrysemys* embryos. Embryos reached stages 9, 12, 15, 19, and 22 after approximately 8, 12, 19, 24, and 26 days of incubation at 26°C, and after approximately 5, 9, 15, 19, and 23 days at 31°C, respectively. Total RNA was extracted from trunks (stage 9), adrenal-kidney-gonadal (AKG) complex (stages 12, 15) and gonads alone (stages 19, 22). Stage 15 was targeted as it demarcates the onset of the thermosensitive period before sex determination and differentiation takes place when important differential transcription occurs in the bipotential gonad of mice [62] and of turtle embryos [42]. Stages 9–12 were targeted as important events occur during this time that shape the development of the genital ridge and which are intimately linked to the developing kidneys [63], and because previous studies by us and others show that temperatures experienced prior to the canonical thermosensitive period influence sex ratios and the transcription of genes underlying sexual differentiation in TSD turtles [25, 28, 31, 34, 35, 42, 64, 65]. RNA was quantified using a NanoDrop® ND-1000 Spectrophotometer, and RNA quality was assessed by the presence of ribosomal bands in agarose gels. RNA-seq libraries were generated using pooled samples from ten embryos per temperature per stage for *Chrysemys* and five for *Apalone* (a single pool per temperature-by-stage). The resulting 20 libraries (1 library/stage/temperature/species x 5 stages x 2 temperatures x 2 species) were sequenced using Illumina’s HiSeq 2000, and ~35 million 100-bp paired-end reads were obtained per library.

### Transcriptome assembly

Reads were splice-mapped across exon boundaries to the *Chrysemys* genome version 3.0.1 [51] using GSNAP (version 2012-03-23), with the novel-splicing feature turned on [66]. Independently, reads were quality filtered and adapter sequences were removed with Trimmomatic [67] and assembled into species-specific *de novo* transcriptomes using the Trinity package (release 2013-02-25) [68] and their quality compared with the genome-guided assemblies. *De novo* transcriptomes were annotated using the Trinotate pipeline [69], mapping the longest open reading frame from each transcript/isoform to the SwissProt protein database [70]. *De novo* transcripts were mapped to 22,380 genes from the annotated *Chrysemys* genome [51] using GMAP (version 2012-03-23) [71]. Unannotated transcripts were mapped to *Trachemys scripta* embryonic transcriptome [72] for comparison using GMAP with default settings. To quantify gene expression levels, the reads from each of the libraries were mapped back to these genes using GSNAP[66]. Then, read-counts for each gene were calculated using HTSeq with the–s (strand-specificity) parameter set to no (which denotes that RNAseq libraries were not strand-specific) [73].

### Gene expression normalization

We implemented a novel normalization procedure for read-counts using R version 2.15.2 [74] independently for each turtle species. We employed a mixed approach that combined normalization by the upper-quartile expression levels [75] with normalization to the housekeeping genes *Transferrin receptor* (*Tfr*) and *hypoxanthine phosphoribosyl transferase 1* (*Hprt1*) (which were constitutively expressed across all stages in both species). This approach permitted validation of the transcriptomic expression levels by comparison to extensive expression data from *Chrysemys* obtained by qRT-PCR of candidate genes from individual embryos, and which were normalized to housekeeping genes during a separate study [42] using independent RNA samples from the present study. To test the effect of the normalization procedure on the number of differentially-expressed genes, we conducted multiple Fisher exact tests between transcript expression levels that were normalized by (1) upper-quartile only (procedure 1, named UQ100), (2) upper-quartile after eliminating the top 1 percentile of transcripts with the highest expression (procedure 2—UQ99), (3) upper-quartile and house-keeping gene normalization (procedure 3—UQHK100), and (4) upper-quartile and house-keeping gene normalization after eliminating the top 1 percentile of transcripts with highest expression (procedure 4—UKHK99).

### Differential expression tests

Differential expression tests were performed per developmental stage between the MPT and FPT for *Chrysemys* (TSD), which correspond to high/low temperature for *Apalone* (GSD). Fisher’s exact tests were used to evaluate differential gene expression between high and low temperature treatments at each developmental stage in each species, as this statistical method permits the analysis of un-replicated samples [75–77]. Briefly, Fisher’s exact test is based on a 2x2 contingency table (Table 2)

**Table 2:** Contingency table for Fisher’s exact test.

	26°C	31°C	Total
**Gene X**	*n*_*11*_	*n*_*12*_	*n*_*11*_ *+ n*_*12*_
**Remaining genes**	*n*_*21*_	*n*_*22*_	*n*_*21*_ *+ n*_*22*_
**Totality of genes**	*n*_*11*_ *+ n*_*21*_	*n*_*21*_ *+ n*_*22*_	*N*

where *n*_*ki*_ is the observed read count for focal gene X (k = 1) or for all other genes in the transcriptome (k = 2) for treatment *i* (*i = 1* for 26°C and *i* = 2 for 31°C); *n*_*11*_ *+ n*_*12*_ is the marginal total for focal gene X; *n*_*21*_ *+ n*_*22*_ is the marginal total for the remaining genes in the transcriptome; *n*_*11*_ *+ n*_*21*_ is the marginal total for the 26°C treatment; *n*_*21*_ *+ n*_*22*_ is the marginal total for the 31°C treatment; *N* is the grand total (reviewed in [76]). This approach tests the null hypothesis that H_0_: θ = 1, where *θ* = *π*_11_*π*_22/_*π*_12_*π*_21_ and where *π*_*ki*_ is the true proportion of counts in cell *k*,*i* (*k* = 1 for focal gene X or 2 for all other genes; *i* = 1 for 26°C and 2 for 31°C). In other words, this approach tests the null hypothesis that the proportion of counts (gene expression) of focal gene X between 26°C and 31°C is the same as for all other genes. It is noted that the lack of replication implies that differences between treatments detected here are not generalizable in the way that inferences from replicated datasets would be, and thus, they should be treated as suggestive hypotheses that warrant corroboration by further analyses. The resulting p-values of Fisher’s exact tests were corrected for false discovery (FDR) [78]. Then, we concentrated on the highly differentially expressed genes meeting a stringent FDR-corrected cutoff of 1e^-10^ chosen arbitrarily. Differentially-expressed genes were annotated using the KEGG database [79]. Enrichment analyses of Gene Ontology (GO) categories against their respective species-specific transcriptomes were conducted using the DAVID Bioinformatics knowledgebase [80]. Additionally, turtle transcriptomes were tested for enrichment against the mouse gonadal transcriptomes [21] using DAVID [79] to test for transcriptional divergence between the turtle and mammalian lineages. In a complementary approach, we randomly subdivided each read library into 2 and 3 subsets (or “subsamples”) [81], identified the GSNAP alignments corresponding to these subsamples, and regenerated read counts per gene. We then used DESeq [82] and EdgeR [83] to independently determine the differentially-expressed genes by leveraging the multiple subsamples while controlling false discoveries at 1%. Finally, the R package WGCNA [84] was used to identify modules of genes co-expressed across turtle embryonic stages in the original set of libraries as well as the subsamples.

## Results

### Transcriptome assembly

RNAseq data was obtained from *Chrysemys* embryonic tissues before the thermosensitive period when the gonads are not yet discernible (stages 9 trunks, stage 12 adrenal-kidney-gonad complexes—AKGs), at the onset of the TSP when gonads are hard to separate from surrounding tissue (stage 15 AKGs), and during the mid and late TSP (stage 19 and 22 gonads), from male-producing temperature (MPT = 26°C), and female-producing temperature (FPT = 31°C) [42]. Identical incubation conditions and sampling scheme were followed for *Apalone*. Such mix-tissue sampling has been used in recent transcriptomic studies of other TSD turtles [25]. *De novo* transcriptome assemblies constructed using Trinity [68] resulted in a high percentage of mapped reads (>92%), and high representation of Core Eukaryotic Genes (CEGs) (>77%) and mammalian urogenital development pathway genes in both species (>96%) (Table 3). All subsequent analyses reported here are based on the *de novo* transcriptome assemblies. We also tested the alternative approach using reference genome-guided assemblies. However this approach was discarded because while the mapping rate of the *Chrysemys* libraries to the *Chrysemys* reference genome [51] was high (97%), that of *Apalone* was only 44% (Table 3) and resulted in significantly fewer gene models for *Apalone* (5,596 unique to *Apalone*, 14,661 unique to *Chrysemys*, and 23,465 overlapping). The problem persisted when using as a reference the genome of *Pelodiscus sinensis* [85], a close relative of *Apalone*, because the *P*. *sinensis* assembled genome is more fragmentary than that of *Chrysemys*, as evidenced by the lack of complete exonic sequences for several genes (such as some homologs of mammalian urogenital genes: *Ctnbb1*—missing part of exon 4; *Wnt4*—missing exon 1 and part of exon 4; *Dmrt1*—exon 1 is mis-assembled/mis-annotated; *Sox9*—exon 1 is mis-assembled).

**Table 3:** Genome-guided and *de novo* transcriptome assembly results for *Chrysemys picta* (CPI) and *Apalone spinifera* (ASP). CEGs = Core Eukaryotic Genes.

		*G*enome-guided	*De novo* (genome-independent)				
Species	Total reads (million)	Million reads mapped (%)	Annotated transcripts (total)	Longest transcriptt (bp)	Percent mapped reads	CEGs mapped out of 456 and (%)	Mammalian sex-differentiation genes mapped out of 27 and (%)
***CPI***	305.9	297.5 (97%)	72,615 (279,903)	18,723	92%	377 (83%)	27 (100%)
***ASP***	373.4	163.6(44%)	76,761 (279,753)	28,965	93%	352 (77%)	26 (96%)

### Normalization schemes to identify differentially-expressed genes per species

**Fig 1:**
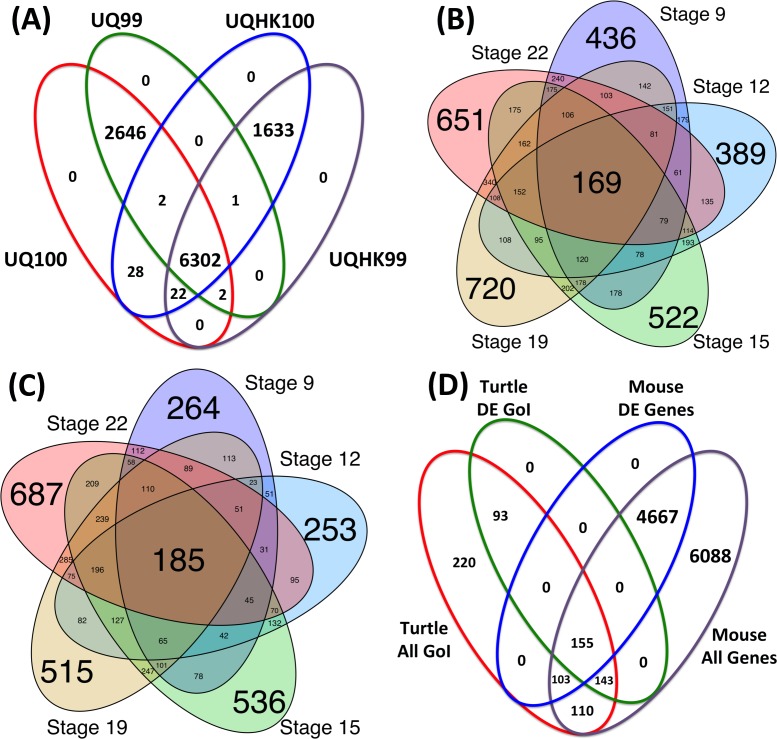
Overlap of differentially-expressed genes by temperature (controlling FDR at 1e-10) for various categories: (**a**) overlap between male- and female-producing temperatures at Stage 22 of *Chrysemys* based on expression levels normalized by (1) upper-quartile (UQ100); (2) upper-quartile excluding the top 1 percentile of transcripts with the highest expression (UQ99); (3) upper-quartile and house-keeping genes (UQHK100); and (4) upper-quartile and house-keeping genes excluding the top 1 percentile of transcripts with highest expression (UQHK99). (**b**) and (**c**): across stages in *Chrysemys picta* and *Apalone spinifera*. (**d**): Overlap of genes of interest present in turtles with the mouse gonadal genes described in Jameson et al., (2012). DE = differentially-expressed, GoI = genes of interest described in Table 5. Differential expression was assessed between temperatures at each developmental stages for each species separately.

**Fig 2:**
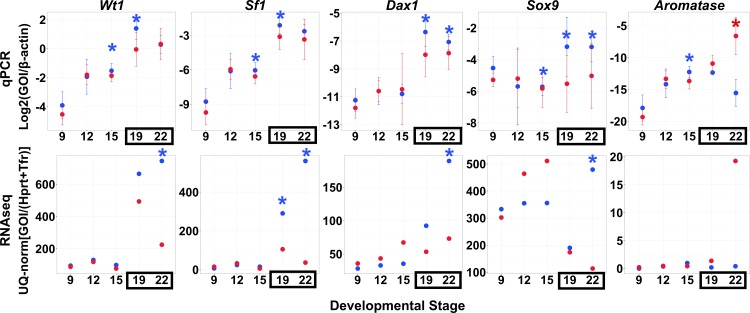
Average qPCR expression at 26°C (blue) and 31°C (red) across developmental stages from qPCR experiments (top panels; modified from Valenzuela et al., 2013) and RNAseq (bottom panels; this study). Mean and one standard deviation are presented. Asterisks denote significant differential expression by temperature. Boxed stages fall within the thermosensitive period.

Gene read abundance was normalized multiple ways, first to the housekeeping genes *Tfr* and *Hprt1* which were constitutively expressed across all developmental stages in both *Chrysemys* and *Apalone*, and then by the standard upper-quartile normalization [75]. The order of these steps did not affect the overall assessment of gene expression. When compared to other normalization procedures described in the methods (UQ100 = upper-quartile only, UQ99 = upper-quartile after eliminating the top 1 percentile of transcripts with the highest expression, UQHK99 = upper-quartile and house-keeping gene normalization after eliminating the top 1 percentile of transcripts with highest expression), the chosen UQHK100 approach resulted in fewer differentially-expressed genes than using the upper-quartile alone (Fig 1A), and therefore, it is a more conservative approach. Furthermore, UQHK100 normalization revealed differential expression patterns which were most consistent with extensive qPCR data of several candidate genes previously obtained for *Chrysemys* using a completely independent set of RNA samples [42], as determined qualitatively by visual inspection of the expression profiles over developmental time for individual genes to identify broadly concordant expression patterns (examining whether differential expression was present and in the same direction per stage and gene between the two studies) (Fig 2). Therefore, we used UQHK100 to identify differentially-expressed genes for further enrichment analyses to ensure unbiased comparisons between species.

### Gene annotation and genes of interest

The longest open-reading frame was used to predict protein sequences, and between 26% and 28% of the transcripts from the *de novo* transcriptome assembly per species were represented in the SwissProt protein database. The *Chrysemys* transcriptome showed an overall higher representation of annotated genes in the *Chrysemys* genome [86] than the *Apalone* transcriptome (Table 4). Of the transcripts not annotated in SwissProt, 252 from *Chrysemys* and 169 from *Apalone* correspond to non-coding RNA sequences (ncRNAs) [87] as identified by BLAST [88]. Among these, 115 out of the 252 transcripts In *Chrysemys* and 70 out of the 169 in Apalone were differentially-expressed (S2 Table). A majority of these ncRNA transcripts were upregulated at 26°C in both species of turtles (*Chrysemys*’ MPT), 36 were upregulated during developmental stages 9, 19 and 22 in *Chrysemys* and another 38 were upregulated only during stages 19 and 22 (*Chrysemys*’ TSP). Of the 25 ncRNA transcripts that overlap, all but 3 show similar differential expression pattern in both species (S2 Table).

**Table 4:** Number of annotated genes identified in the transcriptomes of *Chrysemys picta* (CPI) and *Apalone spinifera* (ASP) by treatment (26°C and 31°C) and developmental stage (9, 12, 15, 19, 22) out of the 22,380 annotated genes present the *Chrysemys picta* reference genome.

		Species			
		*CPI*		*ASP*	
Developmental Stage	Treatment	Genes per treatment	Genes per stage	Genes per treatment	Genes per stage
**Stage 9 (Trunks)**	26°C	13,362	13,687	11,231	11,673
	31°C	13,474		11,205	
**Stage 12 (AKG)**	26°C	13,308	13,714	11,432	11,909
	31°C	13,557		11,517	
**Stage 15 (AKG)**	26°C	13,215	13590	11,182	11,711
	31°C	13,313		11,199	
**Stage 19 (Gonad)**	26°C	13,598	13,742	11,372	11,929
	31°C	13,328		11,507	
**Stage 22 (Gonad)**	26°C	13,182	13,629	11,345	11,988
	31°C	13,398		11,618	
**TOTAL across stages**		13,929		12,667	

AKG = adrenal-kidney-gonadal.

To search for candidates which may be involved in the conversion of temperature signals into sex-specific development and thus may have a significant role in TSD based on their differential expression pattern by temperature, we focused on known genes involved in (a) vertebrate sex determination/differentiation in model mammals and birds [18, 42, 56, 89], (b) epigenetic modification [57], (c) hormonal pathways [58] and (d) general sensing responses [59], out of the variety of annotated genes. These target categories included heat-shock genes, transient receptor potential genes, germ-line and histone-related genes, androgen- and estrogen related genes and genes linked to human/chicken sex chromosomes (http://www.ensembl.org/info/data/ftp/index.html). While the overall composition of the transcriptomes of the two turtle species was similar with regard to these categories, a few genes present in *Chrysemys’* transcriptome were notably absent in *Apalone* across all stages, including some genes X-linked in human and Z-linked in chicken (Table 5). Further, some genes were differentially expressed by temperature in both turtles (S2–S11 Tables). Interestingly, a number of genes that are involved in histone modification show low temperature bias (MPT) just before the onset of the thermosensitive period in *Chrysemys* but high-temperature bias in *Apalone*: histone H1-x like protein (H1x-like), histone chaperone protein (*Asf1B*-like), H3-Histone family 3B (*H3f3b*) and Nuclear autoantigenic sperm protein (*Nasp*). Details about the transcriptional response of these genes of interest are presented in the discussion.

**Table 5:** Number of genes per category of interest present in the transcriptomes of *Chrysemys picta* (CPI) and *Apalone spinifera* (ASP).

Gene category	*CPI*	*ASP*	*Differences*: *ID of genes absent in* *A*. *spinifera transcriptome*
Heat shock	27	27	None
Transient receptor potential	16	16	None
Germ cell-related	**85**	**83**	• ANXA9 • INCA1
Ubiquitin-related	**200**	**192**	• E3-ubiquitin protein ligase TRIM41-like HERC6 • E3 ubiquitin/ISG15 ligase TRIM25-like • E3 ubiquitin-protein ligase TRIM39-like • Ubiquitin carboxyl-terminal hydrolase 8-like • ubiquitin-60S ribosomal protein L40-like • E3 ubiquitin-protein ligase TRIM39-like • ubiquitin-like protein ISG15-like
Kinases	**524**	**513**	• HIPK4 • serine/threonine-protein kinase SBK2-like • myosin light chain kinase smooth muscle-like • pseudopodium-enriched atypical kinase 1-like • ANKK1 • adenylate kinase 8-like • cyclin-dependent kinase 4 inhibitor B-like • ITK • proline-rich receptor-like protein kinase PERK9-like • c-Jun-amino-terminal kinase-interacting protein 3-like • putative uncharacterized serine/threonine-protein kinase SgK110-like
Histone-related	**43**	**37**	• histone H2A type 1-F-like • histone H2A.x-like • histone H2B 8-like-1 • histone H2B 8-like-2 • histone H2A.J-like • histone H2B 7-like
Linked to human h sex chromosomes	**146(1)**	**144(1)**	• FRMD7 • SPRY3
Linked to chickencsex chromosomes	**349(1)**	**345(1)**	• TMEM174 • CER1 • GZMA • SIGLEC15
Cell proliferation	10	10	None
Androgen/Estrogenrelated	19	19	None
Homologs of epigenetic genes	56	56	None

Many of these genes are also differentially-expressed by temperature in both species (see S2–S11 Tables). Gray cells denote categories with some genes absent in the *Apalone spinifera* transcriptome.

### Differential expression in painted turtles (TSD)

The differential expression patterns of five candidate genes previously detected by qPCR [42] and used here for validation of our transcriptomes, were recapitulated by the RNA-seq data when qPCR differences in expression were large. However, smaller expression differences identified by qPCR at certain embryonic stages passed undetected in our RNA-seq transcriptomes (Fig 2). We then chose a highly conservative p-value cutoff value of 1e^-10^ to further correct for false positives in our differential expression tests. This approach revealed significant overlap of highly differentially-expressed genes across developmental stages for both species (Fig 1B and 1C). The same differentially-expressed genes were also recovered with a second approach where reads from each RNAseq library were randomly subdivided into multiple representative subsamples [81] which were then used in the differential expression analysis using both DESeq and EdgeR toolkit [82, 90]. Among these, we identified 1065 genes that were differentially-expressed only in *Chrysemys* across development (S3 Table). Some results of particular interest are highlighted below.

**Fig 3:**
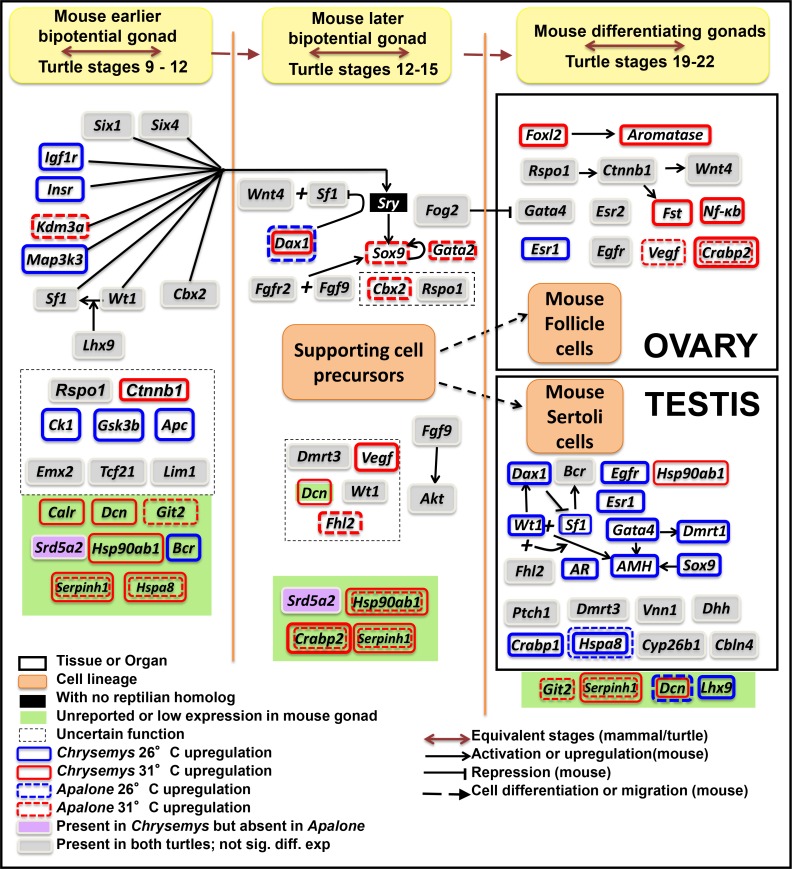
Differential expression in *Chrysemys picta* and *Apalone spinifera* turtles based on RNAseq of a subset of genes involved in the mammalian urogenital pathway and other genes of interest for turtle gonadogenesis. The list of genes and activation or repression information derives from Valenzuela 2008b, Liu et al, 2009, Chassot et al., 2012, Eggers et al., 2014, Lai et al., 2014 plus other sources cited in the text, while information about the timing and pattern of expression correspond to those observed in turtles during the present study. Approximate equivalency is provided between mice and turtle developmental stages of gonadal development. [not sig. diff. exp. = Not significant differential expression].

**Fig 4:**
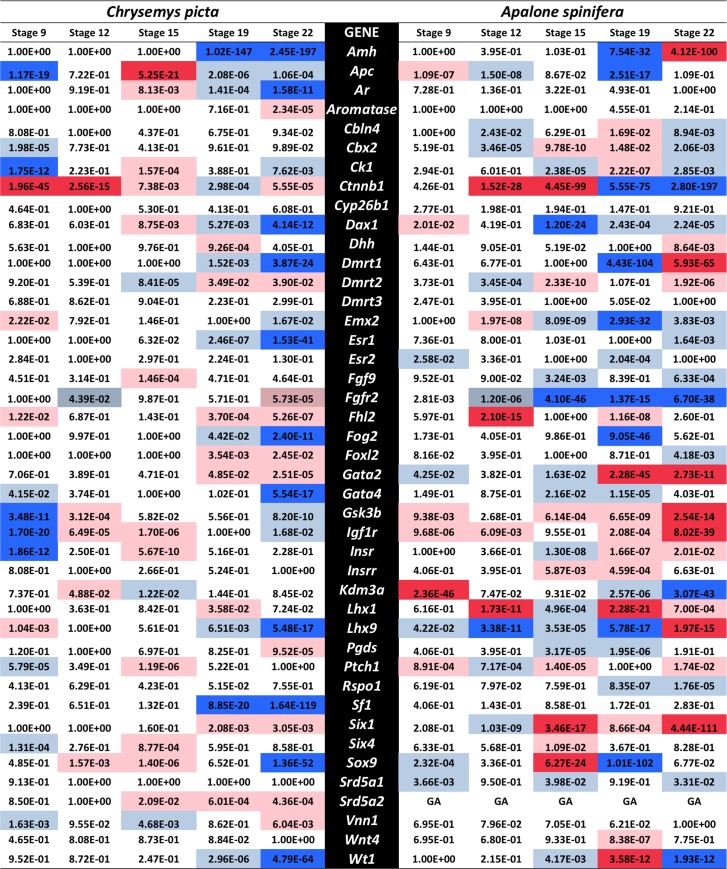
P-values of differentially-expressed genes (after applying Benjamini-Hochberg correction) linked to mammalian urogenital pathways, showing higher expression at 26°C (blue) and 31°C (red). Highly differentially-expressed genes (while controlling false discoveries at 1e-10) identified in dark blue and dark red. Light colored cells denote significance at a standard α = 0.05. GA = gene absent in transcriptome.

**Fig 5:**
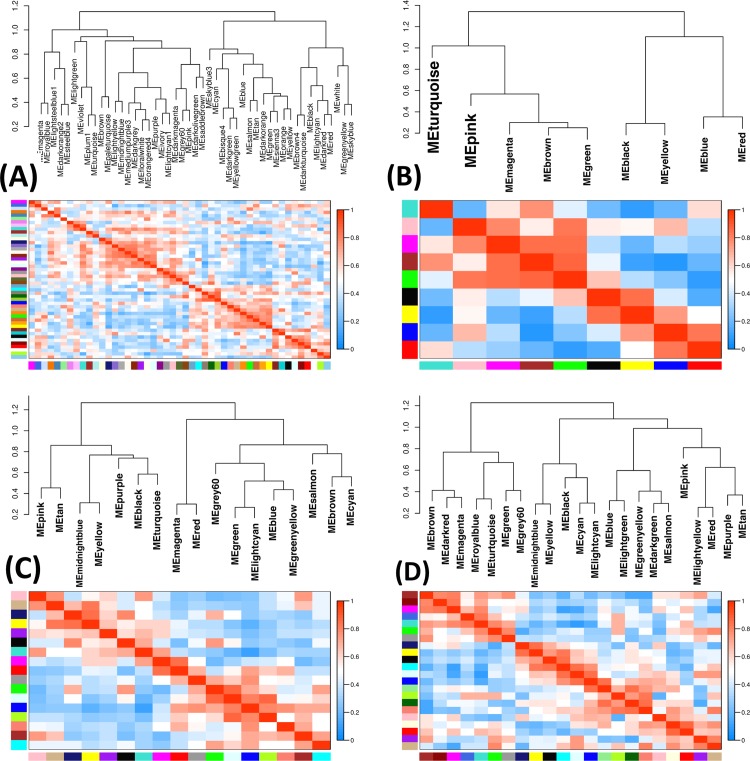
Eigengene networks and dendrograms illustrating the co-expression patterns in developing gonad of (a) *Chrysemys* at 26°C (b) *Chrysemys* at 31°C, (c) *Apalone* at 26°C and (d) *Apalone* at 31°C, of 981 genes of interest described in Table 5. Colors along the x and y-axes represent clusters of genes showing similar expression. Gene order varies by plot along the X and Y-axes.

As mentioned above, our *Chrysemys* RNA-seq data corroborated qPCR results for five known gene homologs involved in gonadogenesis in *Chrysemys*, namely *Sf1*, *Wt1*, *Sox9*, *Aromatase* and *Dax1* [42] which serve to validate our transcriptomes (Fig 2). This approach is similar to the validation level of a recent turtle transcriptome study [91]. RNA-seq was also used to profile a large number of candidate sex-determining genes in *Chrysemys* and *Apalone* (Figs 3 and S2), which were previously uncharacterized in turtles, contributing extensively to our understanding of the full composition of the turtle sex-determining regulatory network. A significant number of these genes in *Chrysemys* show MPT-bias late in the TSP (Stage 22), two (*Igf1r* and *Insr*) before the formation of the bipotential gonad (Stage 9), and only one (β *-catenin*—*Ctnnb1*) shows FPT-bias before the thermosensitive period (Fig 4). Overall, the known vertebrate sex-determination/differentiation network exhibited greater responsiveness to temperature in *Chrysemys* with expression profiles during the thermosensitive period being more concordant with those predicted by the function of these genes in mammals (i.e., genes linked to testicular formation show higher transcription at MPT and those linked to ovarian formation in mammals show higher transcription at FPT in *Chrysemys*, except for *Esr1* and *Hsp90ab1* [Figs 3 and 5]). Contrastingly, expression patterns in *Apalone* were less thermosensitive and more variable, shifting between MPT-bias and FPT-bias throughout development [Figs 3 and 5]. We note that gonads alone were not available from the earlier-stage embryos (we used stage 9 trunks and stage 12/15 AKGs), either because they have not discernibly developed yet (stages 9, 12), or because they cannot be confidently separated without contamination from the surrounding tissue (stage 15). Nonetheless, tissues from these early embryos (which do contain the developing gonad) represent a critical sampling time to detect early responses of key candidate genes to temperature, and their transcriptional profiles revealed that the machinery underlying sexual development is active well before the “canonical” temperature-sensitive period.

### Gene Ontology (GO) Enrichment

At each embryonic stage the differentially-expressed genes were enriched for a number of GO pathways. While no GO pathways were consistently deployed in both turtle species throughout development, GO pathways relating to translation and translational elongation were present across three out of the five stages in both taxa. Overall, we found more shared pathways by stage (including general cell functions such as mitotic cycles, mRNA processing and RNA-splicing) between *Chrysemys* and *Apalone* before stage 15 (onset of TSP in *Chrysemys*) than later in development, suggesting that temperature triggers different network modules after the onset of thermosensitive period in *Chrysemys* than in *Apalone*. Indeed, enriched pathways during stages 9 and 12 in both turtles, including intracellular transport, protein localization and protein catabolic processes remained enriched only in *Chrysemys* after stage 15 (S4 Table). In contrast, genes upregulating protein ubiquitination and ubiquitin protein ligase were enriched only in stage 12 of *Apalone* and not in *Chrysemys*.

### Novel transcripts

Around half of *Chrysemys* (53%, or 150,195/279,903) and *Apalone* transcripts (54% or 152,579/279,753) were absent in SwissProt or ncRNA databases. However, 87% (131,131) of *Chrysemys* transcripts were mapped to the *Chrysemys* genome, of which only 7% (10,660) were unannotated, indicating a gap between the SwissProt/ncRNA databases and the annotated *Chrysemys* genome. Among these, most differentially-expressed transcripts are MPT-upregulated at stages 9, 19 and 22 (S1 Fig). Only 50% of *Apalone* novel transcripts could be mapped to the *Chrysemys* genome and 26% to the *Trachemys scripta* whole-body transcriptome [72], while 68% of *Chrysemys* novel transcripts mapped to *T*. *scripta*. Since this mapping was carried out under low stringency conditions, this difference is likely due to the absence of many novel transcripts in *Apalone*, the GSD turtle, as extensive divergence is not expected in coding sequences between *Chrysemys* and *Apalone* given that transcripts from *Chrysemys* and *Apalone* are more often identical than not (data not shown).

### Gene clustering and co-expression

The genes of interest (Table 5) clustered into modules of co-expression patterns across embryonic stages that differ between turtles. Interestingly, *Chrysemys* showed stronger clustering differences between temperatures, with more gene modules at 26°C (45) than at 31°C (10) (Figs 5A, 5B, S3A and S3B). In contrast, *Apalone* did not differ significantly between temperatures in the number of gene modules (Figs 5C, 5D, S3C and S3D). Similar species-specific clustering differences were also detected for 157 core eukaryotic genes (data not shown). The composition of the largest clusters also varied by temperature in terms of GO pathways (Table 6).

**Table 6:** Categories of GO pathways enriched (at p = 0.05) in the largest co-expressed clusters in the *Chrysemys* and *Apalone* embryonic transcriptomes.

	*Chrysemys picta*		*Apalone spinifera*	
	26°C	31°C	26°C	31°C
**Cluster 1**	Cell proliferation (2)	Immune response (2)	Metabolic process (9) Cell cycle (5)	Cell cycle (3)
**Cluster 2**	• Post translational modification (1) • Signal transduction (1)	Protein translation (1)	Embryonic development (3)	• Cell & organ development (10) • Metabolic process (4)
**Cluster 3**	Post-translational modification (3)	Cell proliferation (2)	Cell proliferation (1)	Cell cycle (2)

## Discussion

The evolution of sex determination remains an evolutionary enigma, yet this developmental process is critical for the production of sex ratios and consequently for the dynamics and evolutionary potential of populations [92]. Turtles are an ideal study system since TSD and GSD co-occur in this group, yet relatively little is known about the gene network controlling turtle urogenital development. Here we present an initial characterization of the full composition of this network using RNAseq and its transcriptional response to incubation temperature in developing gonads of TSD and GSD turtles. This effort sheds light on the genome-wide architecture of vertebrate gonadogenesis and the evolution of its environmental sensitivity in TSD and GSD systems [45–54]. While the expression of some candidate genes has been profiled in a few TSD reptiles (S1 Table) and two transcriptome analyses were recently reported in TSD turtle and alligator [24, 25], *Apalone* remains to our knowledge the only turtle GSD genus [55] whose embryonic urogenital transcription has been studied (this study and [27, 28, 31, 34, 35]. We note that because molecular sexing was unavailable for *Apalone* when data were collected for this study, only temperature effects (and no sex effects) could be analyzed here. Future studies will be able to leverage novel molecular sexing techniques [93] for a more comprehensive test of temperature, sex, and their interaction on transcription in *Apalone*. Overall, our data provides circumstantial evidence that the transcriptional circuitry underlying gonadogenesis in TSD and GSD turtles is broadly the same, and that differences between these mechanisms are largely due to the differential deployment of these common elements as detailed below. We identified novel candidate genes whose early differential expression suggest that they may contribute to transmitting the temperature signal to the developmental pathway, potentially helping determine the sexual fate in TSD turtles, as well as candidate genes undescribed in the gonadal regulatory network of mice and chicken. We first discuss general characteristics of the transcriptomes generated here and then address a series of questions related to our central hypotheses.

### Transcriptome assembly

The genome-guided transcriptome assembly using *Chrysemys* as reference [51] worked well for *Chrysemys* but produced poor results for *Apalone* (44% mapped reads and fewer gene models; Table 3), underscoring the extensive divergence accrued in these turtle genomes since their lineages split >180mya [12]. Using the *Pelodiscus sinensis* genome as reference [85] did not solve this problem, and comparative approaches require common analyses for all species. However, *de novo* transcriptome assemblies had similar high quality and permitted the discovery of novel transcripts previously unidentified in public databases.

Contrastingly, the *Chrysemys* genome [51] was useful to map the transcripts from the *de novo* assemblies (since transcripts are longer and align better than reads) to quantify the representation of annotated genes per library (Table 4). The *Chrysemys* transcriptome had an unsurprising slightly higher representation of annotated genes overall (62%) than *Apalone* (57%). The *P*. *sinensis* genome was excluded here because its annotation is less extensive than *Chrysemys’*. These observations highlight the need to improve current turtle genome assemblies [52], whose re-annotation is aided by RNAseq data, and to sequence additional genomes from representative phylogenetic lineages to illuminate turtle and vertebrate genome evolution and aid future ecological and evolutionary genomic studies.

Using these assembled transcriptomes we address several hypotheses about (1) the composition of the vertebrate gene network governing sexual development in turtles, (2) how it might perceive and transmit environmental temperature inputs, (3) its similarities or differences between TSD and GSD, and (4) whether it may contain reptile-specific elements, as described in the sections below.

**1. The vertebrate gene regulatory network underlying sex determination/differentiation is present and active in TSD and GSD turtles**.

The following paragraphs highlight the transcriptional patterns of known vertebrate determination/differentiation genes found in our transcriptomes (full gene names are presented in Table 1).

### Genes in the vertebrate gonadal network but unknown in turtles

RNAseq provided novel embryonic transcriptional profiles in *Chrysemys* and *Apalone* of several vertebrate genes unstudied in painted and GSD turtles (Fig 4), including genes implicated in testicular differentiation in mammals [*Amh*, *AR*, *Cbln4*, *Dhh*, *Dmrt2*, *Fgf9*, *Fgfr2*, *Fhl2*, *Fog2*, *Pgds*, *Ptch1*, *Srd5a2 and Vnn1*], genes involved in ovarian formation [*Ctnnb1*, *Esr2*, *Foxl2*, *Gata2*, *Rspo1* and *Wnt4*], and genes important for both testicular and ovarian function or general gonadogenesis prior to their sexual commitment [*Cbx2*, *Ck1*, *Gsk3b*, *Apc*, *Insr*, *Igf1r*, *Kdm3a*, *Six1*, *Six4*, *Dmrt3*, *Emx2*, *Esr1*, *Gata4*, *Lhx1*, *Lhx9*] [31, 89].

### Known genes in the turtle gonadal network

Multiple genes involved in turtle sex determination have been studied with candidate gene approaches, including *Vasa*, *Dazl*, *Amh*, *Foxl2*, *Dmrt1*, *Aromatase*, *Androgen receptor* and *Estrogen receptors* α and β, among others [38, 94–97]. Our transcriptomes recapitulated expression patterns from qPCR for five such genes previously reported in *Chrysemys* [27, 28, 31, 34, 35, 98], although subtle differences passed undetected in the transcriptomes (Fig 3), perhaps because transcriptomic inferences have lower power overall than qPCR approaches [43, 44]. We note that to avoid false positive results from the absence of biologically replicated transcriptomes, and from the potential bias introduced by the lower number of embryos per RNA library for *Apalone* compared to *Chrysemys* in our study, we applied a stringent cutoff of 1e-10 to control for false discoveries, and discarded any genes with lower significant differential expression (Fig 5). Taken together, our results highlighted below reveal that the vertebrate gene network regulating primary sexual development is highly conserved in its composition and is active in turtles, but regulated differently between TSD and GSD turtles, and between turtles, mammals and birds (Fig 2, S1 Table).

Importantly, we found that overall, *Chrysemys* exhibits more extensive thermosensitive transcription of the genes in the vertebrate regulatory network than *Apalone*. Also, the expression profiles during the thermosensitive period are fairly concordant with those predicted by the function of these genes in mammals, such that numerous elements involved in mammalian testiculogenesis show higher transcription at MPT and elements involved in mammalian ovariogenesis show higher transcription at FPT in *Chrysemys* (Fig 5). The notable exceptions are *Esr1* and *Hsp90ab1*, which display a transcription pattern opposite of that in mammals, perhaps revealing a new function of these genes in turtles. Such turnovers are not unprecedented as extensive transcriptional evolution has taken place in elements of this network across vertebrates [42]. In contrast, expression patterns in *Apalone* were more variable, shifting between MPT-bias and FPT-bias throughout development, as might be expected under relaxed selection after the evolution of GSD from TSD.

**2**. **How are thermal signals converted to TSD sex-specific development? Does thermal-signal transmission involve the epigenetic machinery, hormonal pathways, and general sensing responses?**

### Differentially-expressed genes by temperature

We searched for genes showing thermosensitive expression in *Chrysemys* (TSD) to uncover candidate temperature sensors or transducers that may activate TSD male and female gonadogenesis. We detected many such genes, including numerous homologs of mammalian sex determination/differentiation genes, plus previously undescribed candidates (Fig 5) as mentioned above. A comparison of differential expression by temperature across select vertebrates is summarized in S1 Table. It is important to note that trunks were examined at stage 9, AKGs at stages 12 and 15, and gonads alone at stages 19, 22, such that gene expression from the developing AK or other organs may contribute to the differences or lack thereof between temperatures at these earlier stages of development. Of the genes in this regulatory network, *Amh*, *Ar*, *Esr1*, *Fog2*, *Gata4* and *Lhx9* show significant MPT (26°C) bias at stage 22 in *Chrysemys* (i.e., during the thermosensitive period when temperature has a strong effect to bias the resulting sex ratios) and are thus important candidate genes for turtle thermosensitive testicular differentiation in TSD vertebrates that deserve further functional research. Noteworthy, our analyses show a consistent FPT (31°C) bias in the expression of the Cold-inducible RNA-binding protein (*Cirbp*) in both *Chrysemys* and *Apalone*. This pattern of temperature-dependent expression in the *Cirbp* gene was shown to help govern ovarian development in the snapping turtle *Chelydra serpentina* (a TSD turtle) via allelic dimorphism, and represents an important novel TSD candidate gene [40]. Combined, our results and those in *Chelydra* indicate that *Cirbp* upregulation at warm temperature might be ancestral to turtles and thus perhaps relict in *Apalone* (as proposed for *Wt1* and *Dax1* [27, 31]). Alternatively, the differential expression of *Cirbp* in *Apalone*, identical to that of *Chrysemys* and *Chelydra*, would call into question its purported key role as a TSD element [40]. We note that in the absence of sex information from the *Apalone* embryos in our study, it remains plausible that some of the thermal effects detected here might be due to sampling effects (e.g., to the predominance of one or the other sex in some of the pools of RNA from *Apalone* used in this study). Further research is warranted using recently developed sexing techniques for *Apalone* [93] to test these alternative hypotheses directly.

### Genes in other functional categories

We explored additional functional gene categories of plausible transducers of the temperature signal to gonadal developmental, some previously known as temperature-sensitive or linked to gonadal formation in other animals [57–59]. These include vertebrate genes involved in gonadal and germ-line differentiation, androgen- and estrogen related genes, and genes linked to sex chromosomes, heat-shock and transient receptor potential genes and histone-related genes. Many of these genes exhibited thermosensitive expression in both turtles (S2–S11 Tables), including genes involved in histone modification, several kinases, genes involved in androgen- and estrogen signaling pathways, sex-linked genes and heat shock proteins. Overall, transcriptome composition was similar between species with some noticeable differences. Namely, *Apalone*’s transcriptome exhibited slightly lower representation of kinases, ubiquitin- and histone-related genes (Table 5), although we confirmed that they are present in the genome of *Apalone* using BLAST. Kinases are indispensable for cell functioning and orchestrate many cellular processes. One of these, the protein kinase *Map3k4*, directly affects *Sry* and *Sox9* expression in bipotential mice gonads, inducing testicular development [99, 100]. Several heat shock proteins show sexually-dimorphic expression in American alligator (TSD), potentially influencing sex determination [59], but only one showed the same pattern in alligator and *Chrysemys* (*Hspb1—*FPT-biased) whereas the heat shock gene *Hsph1* was upregulated at MPT in the alligator and at FPT in *Chrysemys* (stage 12) (S6 Table). Also surprisingly, comparisons of our turtle and the recent alligator transcriptomes [24] revealed only a handful of genes (S6 and S7 Tables) with shared MPT- and FPT-specific expression pattern between alligator and *Chrysemys* [only 31 out of 207 genes reported to be upregulated at FPT, and 43 out of 250 reported to be upregulated at MPT in the alligator were shared with the *Chrysemys* gonadal transcriptome (S6 and S7 Tables)]. The expression pattern of a few genes was similar between the alligator and *Chrysemys* either by sex or by relative temperature. For instance, *Sf1*, *Amh* and *Amhr2* were upregulated at MPT during the TSP in both species [stages 19 (*Sf1*, *Amh*) and 22 (*Sf1*, *Amh* and *Amhr2*) in *Chrysemys*], while *Crabp2* and *Hspb1* were upregulated at FPT in both taxa. Finally, differences in expression were observed in the transient receptor potential gene *Trpc4ap* and *Tex15*, which were both upregulated at MPT in the alligator but not in *Chrysemys*. No other genes in our categories of interest discussed above overlapped between the *Chrysemys* and alligator. These observations support the notion that significant transcriptional evolution has accrued even among TSD regulatory networks [42] and leads to the hypothesis that perhaps numerous temperature-specific responses may be species-specific.

Sex-linked genes such as *Nf2* and *Prdx4* [101, 102] are differentially-expressed by temperature in *Chrysemys* at stage 9. Similarly, we found thermosensitive expression for *Serpinh1*, *Hsp90ab1 and Hspa8* across stages in both turtles (S8 Table) (and potentially relic in *Apalone*) while expression is monomorphic in mouse [21], suggesting their potential turtle-specific role in gonadogenesis. Additional genes differentially-expressed in turtles but at relatively later stages in the mouse gonad [21] include *Ctnnb1*, a known ovarian inducer [89] (which was early acting at 31°C in both *Chrysemys* and *Apalone*) and *Git2* (a sex-linked gene in *Pelodiscus sinensis* [101]), among others (Fig 1D, S3 and S16 Tables). This indicates that genes already important for gonadal formation at downstream stages in mammals, such as *Ctnnb1*, have been recruited for an earlier than anticipated temperature-specific response in turtles, underscoring the extensive ontogenetic evolution that has accrued in the transcriptional patterns of this regulatory network in vertebrates in general and in turtles in particular [25, 42].

We also identified numerous differentially-expressed ncRNAs (S2 Table) by temperature, which interestingly, exhibited upregulation at the colder temperature (26°C) in the majority of cases and in both turtles, although few ncRNAs transcripts overlapped between species. The biological function of these ncRNAs remains an open question worthy of future study.

### Thermosensitive response of signaling pathways

Distinct cell types may derive from a handful of cell signaling pathways [103]. We found evidence that numerous signaling pathways are differentially regulated by temperature. For instance, *Jak-Stat* signaling, involved in cell proliferation and hematopoiesis [104] exhibits male-bias of *Egfr* expression at stage 22 in *Chrysemys*. Nf-κB signaling plays a role in immune and stress response [105], and involves members of the hypoxia-induced *Tumour necrosis family (Tnf)* [106] and *Breakpoint cluster region* (*Bcr)* [107] which showed female-bias in *Chrysemys* at stage 22 and male-bias at stage 9, respectively. The receptor gene *Vegf*, which regulates sex-specific gonadal vasculogenesis [108] was also female-biased at stage 15 in *Chrysemys* and high-temperature biased during stages 19–22 in *Apalone*. Further, retinoic acid has been identified to induce meiosis in mice germ cells regulating ovarian formation [109]. Two retinoic acid binding proteins were differentially transcribed: *Crabp1* (male-biased during *Chrysemys* TSP) and *Crabp2* (female-biased pre-TSP and during the TSP in *Chrysemys*, and upregulated at high-temperature from stage 15 onwards in *Apalone*). Among the signaling pathways implicated in vertebrate sex determination, *Foxl2* and members of the *Wnt* signaling pathway regulate ovarian formation [110, 111]. *Wnt* activates *Ctnnb1*, which inhibits *Sf1* from activating *Sox9* and inducing testiculogenesis [112]. The canonical *Wnt* machinery including *Ck1*, *Apc*, and *Gsk3* show male-bias during stage 9 in *Chrysemys*, and monomorphic expression in *Apalone*, indicating that *Wnt* signaling is active in TSD and GSD turtles, but deployed differentially by temperature. Members of the *Mapk* signaling family, required for *Sry* activation testiculogenesis in mice [100] were also low-temperature biased in turtle bipotential gonads despite the absence of *Sry* (*Map3k3* at stage 9 in *Chrysemys*; *Map3k7* at stage 12 in *Apalone*) (S9 Table), rendering them additional candidates for functional tests. *Akt* signaling is directly activated by *Fgf9* in mice, promoting steroidogenesis (Lai *et al*. 2014), but neither gene showed thermosensitive expression. Finally, *Ras*-mediated signaling is implicated in sex myoblast migration in nematodes [113], and a subtle thermosensitive expression was detected in *Chrysemys* (S9 Table). Thus, *Jak-Stat*, Nf-κB, retinoic acid, *Wnt*, *and Mapk* signaling are potentially involved in TSD gonadogenesis, while this process appears independent of *Akt* and *Ras*-mediated signaling.

**3**. **What makes gonadal networks sensitive to environmental temperature in TSD and not in GSD?**

### Gene enrichment analysis

Enrichment analyses of Gene Ontology (GO) categories represented in the transcriptomes using DAVID [80] revealed that the two turtle species examined here shared more pathways before stage 15 overall, except for chromatin organization and chromatin modification pathways which were enriched only at stage 9 in *Chrysemys*, and pathways linked to protein ubiquitination and ubiquitin protein ligases which were enriched only at stage 12 in *Apalone*. Ubiquitination is a post-translational modification that results in protein degradation [114]. This suggests that temperature triggers a different set of downstream pathways in *Chrysemys* than in *Apalone* potentially leading to sexual fate determination by temperature in the former. Pathways including intracellular transport, protein localization and protein catabolic processes, were enriched in both species at stages 9 and 12, but remain enriched only in *Chrysemys* after stage 15 (S4 Table). Thus, important evolutionary changes may have occurred in GSD turtles in the machinery underlying gonadogenesis preceding the thermosensitive period of TSD turtles (before stage 15), perhaps inactivating genes regulating the male- and female-specific TSD pathways and consequently, determining sex independent of temperature. These and earlier findings (reviewed in [42]) underscore that key thermosensitive events for sexual development occur in early embryogenesis and deserve further research.

Finding differential expression prior to the onset of the canonical thermosensitive period in *Chrysemys* is of particular importance as any such gene may be the key TSD master element that senses the environmental temperature signal or a key activator of the thermosensitive period. Additionally, the disruption of these potential TSD master elements could lead to GSD evolution. Notably, *Ctnnb1* (β-catenin, a member of the *Wnt* signaling pathway) showed female-bias at stages 9 and 12 in *Chrysemys* consistent with its involvement in early ovarian formation in mammals [115, 116]. Similarly, *Ctnnb1* shows high-temperature bias in *Apalone* during stages 12 and 15 but not at stage 9. *Follistatin* (*Fst*) a gene activated by *Ctnnb1* in mice bipotential gonads (Eggers *et al*. 2014) showed slight female-bias (α = 0.05) in *Chrysemys* at stage 9, and slight high-temperature bias in *Apalone* at stage 15 (α = 0.05), suggesting that *Ctnnb1* could also activate *Fst* in turtles. Further, this results indicates that *Ctnnb1* and *Fst* thermosensitive expression may be ancestral to cryptodiran turtles (the suborder to which *Chrysemys* and *Apalone* belong), which if true, would indicate relic thermosensitivity in *Apalone*, and underscores that downstream elements may be key to rendering GSD immune to temperature as is the case of *Wt1* and *Dax1* whose thermoresponsive activity is rendered ineffective via the loss of thermosensitivity of *Sf1* [27, 31]. Indeed, *Ctnnb1* is a repressor of *Sf1* [112], a gene with thermosensitive expression in *Chrysemys* but not *Apalone* [28]. Also noteworthy, *Chrysemys* (and not *Apalone*) shows upregulation at MPT of *Insr* and *Igf1r* during stage 9, two genes indispensable for testiculogenesis in mice [117] which antagonize the *Ctnnb1-Wnt* signaling pathway essential for ovarian formation. This indicates that the same molecular antagonism exists in TSD turtles, is active before the canonical thermosensitive-period, and could influence growth trajectories via the insulin receptor family, inducing male determination [118] and other sexual dimorphisms with potential temperature-specific fitness consequences [119]. These early differences between TSD and GSD systems may have functional significance for the evolution of sex determination.

### Comparison with the slider turtle *Trachemys scripta*

**Fig 6:**
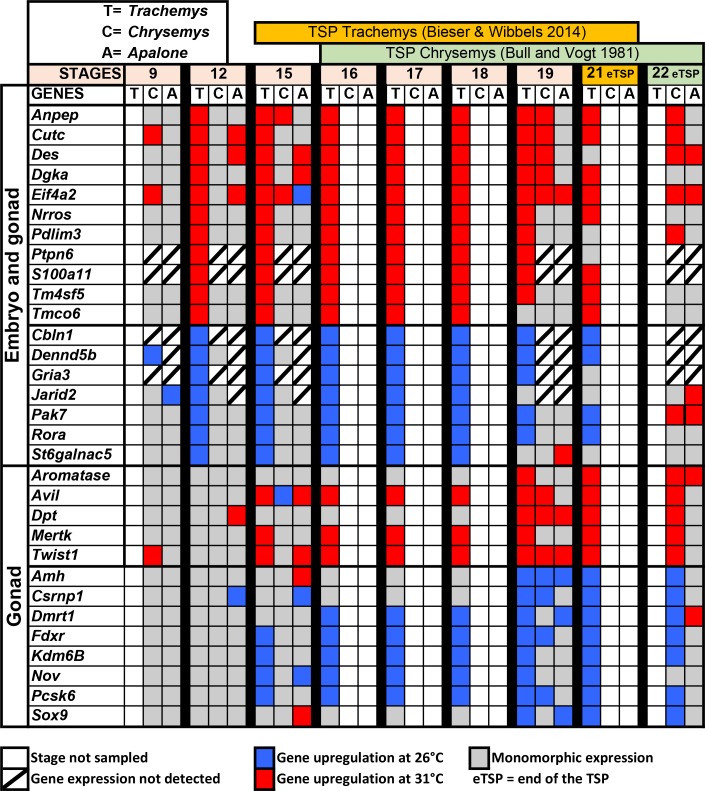
Comparative transcriptional patterns in *Chrysemys*, *Apalone* and *Trachemys* for a subset of common elements in the regulatory network of sexual development across embryonic stages at male- (low) and female- (high) producing temperatures for the TSD taxa.

Both *Trachemys* and *Chrysemys* have TSD and diverged near 30 million years ago [120]. qPCR evidence exist that transcriptional patterns of some of genes involved in sexual development have diverged between these two species during this time [42], but the extent of differences and similarities in other elements of this network remains unclear. Here we compared the expression stage-by-stage of key common elements between our transcriptomes and the recent *Trachemys* transcriptome [25] (Fig 6). We observed similarities in the expression of certain genes between species, in terms of upregulation at MPT or FPT in both the embryo and gonad simultaneously, or in the gonad alone (Fig 6). It should be noted that the *Trachemys* study did not sample stage 9 as in our study, whereas stages 17 and 18 were examined in *Trachemys* and not in our study. Additionally, the thermosensitive period differs slightly between *Trachemys* (stages 15–21) [121] and *Chrysemys* (stages 16–22) [46], such that while numerically different, both studies contain data from stages at the onset and at the end of the thermosensitive period that can be compared (Fig 6). Differential transcription of numerous genes in this subnetwork was observed in *Trachemys* at stages 12 and 15 that were not observed in *Chrysemys* either because the timing of their expression is divergent, or because they passed undetected by our unreplicated transcriptomic approach, yet when expression was detected the pattern was concordant between species except for *Avil* (Fig 6). Greater similarities were observed later in development in both the embryo and gonad during stage 19 at FPT (*Anpep*, *Cutc*, *Des*, *Dgka* and *Eif4a2*). Among genes that were not differentially expressed during stage 12 but showed MPT or FPT upregulation at stage 15 or later (and thus are candidates for a role as temperature-triggered genes), similar profiles were observed late in the TSP for *Aromatase*, *Avil*, *Dpt*, *Mertk*, and *Twist1*, all of which were upregulated at FPT during stage 21 in *Trachemys* and stage 22 in *Chrysemys*. Genes upregulated at MPT in both turtles at those same stages include *Amh*, *Csrnp1*, *Dmrt1*, *Fdxr*, *Kdm6b*, *Pcsk6* and *Sox9*. Elements with common expression patterns in *Chrysemys* and *Trachemys* are candidate key TSD elements for the Emydidae family of turtles to which they belong. A few genes were also differentially transcribed in *Apalone* late in development, and represent either genes that are thermosensitive in turtles but not involved in TSD, or TSD elements that retained relic thermosensitive expression during GSD evolution. Such genes include *Des*, *Eif4a2* (both upregulated at 31°C during stage 22 in *Apalone*), *Dpt* and *Twist1* (upregulated at 31°C during stage 19).

### Clues to TSD from the comparison of turtle versus crocodilian transcriptomes

Surprisingly few differentially-expressed genes were found in common in the categories of interest between *Chrysemys* (this study) and alligator transcriptomes [24], but the ones detected provide an important insight. Namely, during the TSP of both species *Sf1*, *Amh* and *Amhr2* were upregulated at MPT, whereas *Crabp2* and *Hspb1* were upregulated at FPT. Thus, these genes display a sex-specific expression pattern, i.e., upregulation at MPT or FPT across taxonomic groups regardless of relative temperature given that in *Chrysemys* colder temperatures induce males and warmer values induce females, whereas the opposite is true in alligator [the colder values used in [24] produce females and the warmer temperature produces males]. On the other hand, the heat shock gene *Hsph1* was upregulated at MPT in alligator and at FPT in *Chrysemys*. *Hsph1* therefore, displays a temperature-specific pattern of expression (upregulation at warmer temperature in both species) irrespective of the sex-produced. These observations point to *Sf1*, *Amh* and *Amhr2*, *Crabp2* and *Hspb1* as key common elements in reptilian-TSD for sex-specific development. This finding is consistent with previous research that identified them as critical members of the vertebrate regulatory network for gonadogenesis, and critical during TSD and GSD evolution [e.g., *Sf1* [28, 42], *Amh* and *Amhr2* [122–124], *Crabp2* [125, 126].

### Detection of temporally co-expressed gene clusters

Genes of interest (described in Table 5) clustered during embryogenesis by their co-expression patterns in both turtles. *Chrysemys* differ more in the number of coexpressed modules (45 modules at 26°C; 10 at 31°C) than *Apalone* (16 modules at 26°C, 21 at 31°C) (Fig 5), a pattern similar to core eukaryotic genes used as negative control. This suggests that temperature differentially orchestrates gene co-expression in TSD versus GSD, such that *Chrysemys’* response is more compartmentalized, and *Apalone*’s is broader. However, tests with sexed *Apalone* embryos are needed to rule out any sampling effects that might have contributed to these differences. Some vertebrate sex determination/differentiation genes were clustered in turtles (such as *Cbx2* and *Dmrt2* in *Chrysemys* at both temperatures, and *Ar/Lhx9* and *Insr/Srd5a1* in *Apalone*), whereas no associations among these genes are known in mammals or birds. Future functional assays on these candidates are warranted. Cluster composition differed by temperature and species, as clusters were enriched in different biological pathways, reflecting temperature effects on gene co-expression and the existence of modules in the urogenital network. *Chrysemys* male transcriptomes were enriched for pathways regulating transcription, cell proliferation, reproductive development and amino acid phosphorylation (a kinase activity that has been linked to *Sry* regulation in mice [99, 100]). Immune response functions like lymphocyte and leukocyte activation were female-bias concordant with humans [127]. Cell proliferation, which showed thermosensitive responses here (Table 6, S10 Table), is linked to mammalian sexual development as it is affected by *Sry* and MAPK signaling [128].

### Novel transcripts

A high percentage of novel transcripts in *Chrysemys* (53%) and *Apalone* (54%) are currently uncharacterized in SwissProt (which contains manually curated, non-redundant eukaryotic protein sequences) and the ncRNA databases, corroborating genes that were annotated as “predicted” *in silico* in the *Chrysemys* genome [51]. Many novel transcripts are male-biased at stages 9, 19 and 22 of *Chrysemys* (S1 Fig). In conjunction with the greater number of co-expressed clusters discovered at 26°C, this discovery of higher number of novel *Chrysemys* transcripts at 26°C is indeed curious, and the function of these transcripts merits further investigation.

### New insights on known turtle regulators previously studied in GSD turtles

Our RNAseq data shed new light on some important candidate genes previously studied in turtles as follows.

***Wt1*** regulates the formation of the bipotential gonad, and the maintenance of Sertoli cells and seminiferous tubules in developing testis [129]. *Wt1* is upregulated at low temperature before and during the thermosensitive period (TSP) in *Chrysemys* [42], and in *Apalone mutica* (GSD) (relic thermal sensitivity in GSD turtles) ([27] and this study) at stage 22 (Fig 5). In contrast, *Wt1* expression in mice and chicken gonads is sexually monomorphic through embryogenesis [21, 130]. Finding differential expression from stage 19–22 *Apalone* gonads is important because previous studies in *A*. *mutica* used adrenal-kidney-gonad complexes (AKGs) [27, 28, 31, 34, 35], and expression from the adrenal-kidney can mask gonadal expression, as occurs in *Chrysemys* and other turtles [42, 131–133].

***Sf1*** is required for gonadal and adrenal gland formation and steroidogenic activity [134]. *Sf1* is directly activated by *Wt1*, and its expression is thermo-insensitive in GSD turtles ([28] and this study) as in in *Chelydra* (TSD) [41], but male-biased in *Chrysemys* ([42] and this study) as in the slider turtle *Trachemys scripta* (TSD) [135]. This underscores that *Sf1* expression is evolutionarily labile, as observed across major vertebrate lineages (reviewed in [42]).

***Dax1*** is involved in mammalian ovarian and testicular formation [136, 137]. *Dax1* is upregulated at low temperature in *Chrysemys* ([42] and his study), and in *Apalone* (this study), as observed in *A*. *mutica* [31] perhaps driven by the relic thermosensitive expression of its activator (*Wt1*). In contrast, *Dax1* expression is female-biased in birds and monomorphic in several TSD taxa including the green sea turtle *Lepidochelys olivacea*, *Chelydra* and *Trachemys* (reviewed in [42]).

***Sox9*** is activated by *Sry* in eutherian mammals, tipping the bipotential gonad towards the male fate. *Sox9* is upregulated in *Chrysemys* ([42] and this study). In contrast, *Sox9* in *Apalone* shifts from upregulation at high-temperature (stage 15) to upregulation at low-temperature (stage 19), perhaps reflecting the evolutionary drift in GSD turtles from its ancestral thermal response. Consistently, *Sox9* expression in *A*. *mutica* is monomorphic [34].

***Aromatase*** is key in ovarian formation and steroidogenic activity [138, 139]. *Aromatase* is upregulated at FPT during *Chrysemys’* late-TSP ([42] and this study). *Aromatase* expression is evolutionarily labile across vertebrates [140, 141]. The monomorphic *aromatase* transcription in the bipotential gonad (stages 9 and 12) in turtles observed here is consistent with its expression in chicken [23].

***Dmrt1*** regulates sexual development in vertebrates [56] and its molecular evolution is associated with transitions in sex determination in reptiles [126]. *Dmrt1* is sex-linked in fish [142] and in birds [143]. *Dmrt1* is upregulated in gonads at MPT in *Chrysemys* (this study; and counter to AKG expression [34]), underscoring *Dmrt1*’s critical role in testicular development as in *Trachemys* (TSD) [37]. *Dmrt1* in *Apalone* shifts from low-temperature upregulation (stage 19) to high-temperature upregulation (stage 22) in gonads (this study), whereas its expression is monomorphic in AKG of *A*. *mutica* [34], perhaps also reflecting drift of gene expression during GSD evolution as suggested for *Sox9* [34]. *Dmrt1* expression is male-biased in fish, birds and mammals [23, 42].

**4**. **Are there unique reptilian regulators not present in the gonadal network of other vertebrates?**

### Discovery of new elements in the vertebrate gonadal network

Our dataset also revealed genes expressed in turtle gonads but unreported in mice, thus possibly unique to reptilian (or turtle) gonadogenesis. Among these are *Calr* (female-biased at stage 9) and *Dcn*, a component of the extracellular matrix uncharacterized in the mouse gonad [144], and which showed female-bias in *Chrysemys* stage 15 onwards. These, as well as the differentially-expressed ncRNAs and unannotated novel transcripts identified here (S2 Table, S1 Fig) in both turtles, represent novel candidates for further study. Some of the differentially-expressed ncRNAs identified here are particularly intriguing, such as 7SK RNA and those classified as putative conserved noncoding region given that these ncRNAs can play key roles in transcriptional regulation [87, 145]. The observed higher expression at FPT of *Xist* RNA during the TSP of *Chrysemys* (stage 19), and its absence at other stages and in *Apalone* is also intriguing. Namely, this is the first report of *Xist* expression during gonadal development in turtles lacking sex chromosomes [45], a ncRNA region that is critical for dosage compensation via epigenetic inactivation of the X chromosome in human or its upregulation in *Drosophila* [87, 146].

Finally, genes enriched in hypoxia tolerance and mitochondrial functions that mediate the adaptation to sub-zero temperatures were differentially-expressed in *Chrysemys* across all stages [147], including translocases that function as chaperones across the mitochondrial membranes, the SLC25 family of mitochondrial transporters [148], *Ep300*, *Casp1* and *Thioredoxin* family involved in hypoxia signaling [149, 150]. Our data indicate that these genes, which underlie thermal adaptation, are also active from early development.

## Conclusion

Ours is the first comparative transcriptomic analysis of TSD vertebrates and GSD turtles. Our analyses uncovered numerous homologs of mammalian urogonadal genes that were unidentified in turtles, alongside many turtle-specific novel transcripts. The strengths of the transcriptomic time series through embryogenesis in two species permitted an initial and simultaneous characterization of the genome-wide network underlying gonadogenesis and its response to environmental temperature in TSD and GSD systems. Because a single pool of RNA was available per temperature-by-stage, our inferences should be taken as a strongly suggestive initial glimpse of the genome-wide composition and regulation of the gene network underlying sexual development in TSD and GSD turtles. These results, accompanied by the discovery of previously unknown gonadal regulators in vertebrates that are active well before the onset of the thermosensitive period highlight the value of targeted investigations of early orchestrators of embryogenesis. Further, the thermosensitive response of key elements of multiple signaling pathways potentially governing sex determination observed here, underscore that differences between TSD and GSD in turtles are less likely due to unique elements in this network (although some candidates were identified) but instead, perhaps largely due to the differential deployment of common elements and modules. Our work thus contributes to deciphering the evolutionary puzzle of vertebrate sex determination, and provides significant genomic resources and working hypotheses to guide further investigations in this active field of research.

## Supporting information

S1 Fig Novel transcripts from *Chrysemys picta* that are highly differentially-expressed (while controlling false discoveries at 1e-10). Blue: upregulated at 26°C, red: upregulated at 31°C.
(PDF)

S2 Fig RNA-seq expression patterns for multiple genes previously implicated in mammalian urogonadal development across developmental stages 9–22 at 26°C (blue) and 31°C (red) in *Chrysemys picta* and *Apalone spinifera*. Statistically significant differences are indicated with an asterisk (*). Thermosensitive period is indicated by a box.
(PDF)

S3 Fig Gene co-expression patterns by temperature for each turtle species from the RNA-seq data. Panels a-d illustrate modules of high (red) and low (yellow) co-expression for 981 genes of interest (described in Table 5) profiled across five developmental stages in *Chrysemys picta* [(a) and (b)] and *Apalone spinifera* [(c) and (dc)] at 26°C and 31°C respectively.
(PDF)

S1 Table Genes involved in sex determination–a comparison across reptiles and mammals. (XLSX)

S2 Table Differential expression pattern of transcripts characterized as ncRNA. (XLSX)

S3 Table Comparisons of genes of interest (described in Table 5) between turtle and mouse gonad (Jameson et al., 2012): Column A: Differentially-expressed genes of interest shared between both mouse and turtle; Column B: Differentially-expressed genes of interest in turtle but not present in mouse gonad, Column C: Differentially-expressed in mouse but not differentially-expressed in turtle, Column D: Differentially-expressed in turtle but not differentially-expressed in mouse, Column E: Differentially-expressed in *Chrysemys* but not in *Apalone*, Column F: Differentially-expressed in *Apalone* but not in *Chrysemys*. (XLSX)

S4 Table List of GO pathways enriched in differentially-expressed genes in the turtles. (XLSX)

S5 Table Genes whose embryonic gonadal expression was explored in this study (symbols and names). (XLSX)

S6 Table Expression of genes showing FPT-bias in *Alligator mississippiensis* gonadal transcriptome (Yatsu et al., 2016) in *Chrysemys* and *Apalone*. (XLSX)

S7 Table Expression of genes showing MPT-bias in *Alligator mississippiensis* gonadal transcriptome (Yatsu et al., 2016) in *Chrysemys* and *Apalone*. (XLSX)

S8 Table List of differentially-expressed genes by category—Heat-shock related. (XLSX)

S9 Table List of genes differentially-expressed by signaling pathway. (XLSX)

S10 Table Gene enrichment in the 3 biggest clusters at p = 0.05 according to expression levels by species and temperature. The three biggest modules at each condition are indicated by turquoise, blue and brown colors in that order in S8 Fig.
(XLSX)

S11 Table List of differentially-expressed genes by category–sex-linked genes in vertebrates. (XLSX)

S12 Table List of differentially-expressed genes by category—Histone related. (XLSX)

S13 Table List of differentially-expressed genes by category—Ubiquitin related. (XLSX)

S14 Table List of differentially-expressed genes by category—Androgen/Estrogen related. (XLSX)

S15 Table List of differentially-expressed genes by category—Transient receptor potential related. (XLSX)

S16 Table List of differentially-expressed genes by category—germ cell related. (XLSX)

S17 Table List of differentially-expressed genes by category—cell proliferation related. (XLSX)

S18 Table List of differentially-expressed genes by category—kinases. (XLSX)

S19 Table List of differentially-expressed genes by category—RNA Binding Proteins. (XLSX)

S20 Table Differential expression pattern of genes not characterized in the mouse gonad (Jameson et al., 2012). (XLSX)

## References

[1] Bachtrog D, Mank JE, Peichel CL, Kirkpatrick M, Otto SP, Ashman T-L, et al Sex determination: Why so many ways of doing it? PLoS Biol. 2014;12(7):e1001899 10.1371/journal.pbio.1001899 24983465 PMC4077654

[2] Valenzuela N, Adams DC, Janzen FJ. Pattern does not equal process: exactly when is sex environmentally determined? Am Nat. 2003;161:676–83. 10.1086/368292 12776892

[3] Valenzuela N, Lance VA, eds. Temperature dependent sex determination in vertebrates. Washington, DC: Smithsonian Books; 2004.

[4] Bull JJ. Evolution of sex determining mechanisms. 1983:173–84.

[5] Deeming DC, Ferguson MWJ. Physiological effects of incubation temperature on embryonic development in reptiles and birds In: Deeming DC, Ferguson MWJ, editors. 2004: Cambridge University Press; 1991 p. 147–71.

[6] Rhen T, Schroeder A. Molecular mechanisms of sex determination in reptiles. Sex Dev. 2010;4(1–2):16–28. 10.1159/000282495 20145384 PMC2918650

[7] Tree of Sex Consortium, Ashman TL, Bachtrog D, Blackmon H, Goldberg EE, Hahn MW, et al Tree of Sex: A database of sexual systems. Sci Data. 2014;1. 10.1038/sdata.2014.15 PMC4322564 25977773

[8] Sarre SD, Georges A, Quinn A. The ends of a continuum: genetic and temperature-dependent sex determination in reptiles. Bioessays. 2004;26(6):639–45. 10.1002/bies.20050 15170861

[9] Shine R, Elphick MJ, Donnellan S. Co-occurrence of multiple, supposedly incompatible modes of sex determination in a lizard population. Ecol Lett. 2002;5:486–9.

[10] Holleley CE, O’Meally D, Sarre SD, Marshall Graves JA, Ezaz T, Matsubara K, et al Sex reversal triggers the rapid transition from genetic to temperature-dependent sex. Nature. 2015;523:79–82. 10.1038/nature14574 26135451

[11] Organ CL, Janes DE. Evolution of sex chromosomes in Sauropsida. Integr Comp Biol. 2008;48(4):512–9. 10.1093/icb/icn041 21669812 PMC4553705

[12] Valenzuela N, Adams DC. Chromosome number and sex determination co-evolve in turtles. Evolution. 2011;65 1808–13. 10.1111/j.1558-5646.2011.01258.x 21644965

[13] Sabath N, Itescu Y, Feldman A, Meiri S, Mayrose I, Valenzuela N. Sex determination and the birth and death of species. Ecology and Evolution. 2016; 10.1002/ece3.2277 PMC4984498 27551377

[14] Pokorná MJ, Kratochvíl L. What was the ancestral sex‐determining mechanism in amniote vertebrates? Biological Reviews. 2016. 10.1111/brv.12156 25424152

[15] Mitchell NJ, Janzen FJ. Temperature-Dependent Sex Determination and Contemporary Climate Change. Sex Dev. 2010;4(1–2):129–40. 10.1159/000282494 20145383

[16] Neuwald JL, Valenzuela N. The lesser known challenge of climate change: Thermal variance and sex-reversal in vertebrates with temperature-dependent sex determination. PLoS ONE. 2011;6(3):10.1371/journal.pone.0018117 PMC3063247 21448294

[17] Grayson KL, Mitchell NJ, Monks JM, Keall SN, Wilson JN, Nelson NJ. Sex ratio bias and extinction risk in an isolated population of tuatara (*Sphenodon punctatus*). PLoS ONE. 2014;9(4). 10.1371/journal.pone.0094214 PMC3979778 24714691

[18] Mizoguchi BA, Valenzuela N. Ecotoxicological perspectives of sex determination. Sex Dev. 2016; 10.1159/000444770 27022970

[19] Liu CF, Liu C, Yao HHC. Building pathways for ovary organogenesis in the mouse embryo. Curr Top Dev Biol. 2010;90:263–90. 10.1016/S0070-2153(10)90007-0 20691852 PMC3400115

[20] Wainwright EN, Wilhelm D. The game plan: Cellular and molecular mechanisms of mammalian testis development. Organ Dev. 2010;90:231–62. 10.1016/S0070-2153(10)90006-9 20691851

[21] Jameson SA, Natarajan A, Cool J, DeFalco T, Maatouk DM, Mork L, et al Temporal transcriptional profiling of somatic and germ cells reveals biased lineage priming of sexual fate in the fetal mouse gonad. PLoS Genet. 2012;8. 10.1371/journal.pgen.1002575 PMC3305395 22438826

[22] Munger SC, Natarajan A, Looger LL, Ohler U, Capel B. Fine time course expression analysis identifies cascades of activation and repression and maps a putative regulator of mammalian sex determination. PLoS Genet. 2013;9. 10.1371/journal.pgen.1003630 PMC3708841 23874228

[23] Ayers KL, Davidson NM, Demiyah D, Roeszler KN, Gruetzner F, Sinclair AH, et al RNA sequencing reveals sexually dimorphic gene expression before gonadal differentiation in chicken and allows comprehensive annotation of the W-chromosome. Genome Biol. 2013;14(3). 10.1186/gb-2013-14-3-r26 PMC4053838 23531366

[24] Yatsu R, Miyagawa S, Kohno S, Parrott BB, Yamaguchi K, Ogino Y, et al RNA-seq analysis of the gonadal transcriptome during *Alligator mississippiensis* temperature-dependent sex determination and differentiation. BMC Genomics. 2016;17(1):1. 26810479 10.1186/s12864-016-2396-9 PMC4727388

[25] Czerwinski M, Natarajan A, Barske L, Looger LL, Capel B. A timecourse analysis of systemic and gonadal effects of temperature on sexual development of the red-eared slider turtle Trachemys scripta elegans. Dev Biol. 2016. 10.1016/j.ydbio.2016.09.018 27671871

[26] Spotila LD, Spotila JR, Hall SE. Sequence and expression analysis of WT1 and Sox9 in the red-eared slider turtle, *Trachemys scripta*. J Exp Zool. 1998;284:417–27. 10.1002/(sici)1097-010x(19980801)281:5<417::aid-jez7>3.0.co;2-r 9662829

[27] Valenzuela N. Relic thermosensitive gene expression in a turtle with genotypic sex determination. Evolution. 2008;62(1):234–40. 10.1111/j.1558-5646.2007.00279.x 18053078

[28] Valenzuela N, LeClere A, Shikano T. Comparative gene expression of steroidogenic factor 1 in *Chrysemys picta* and *Apalone mutica* turtles with temperature-dependent and genotypic sex determination. Evol Dev. 2006;8(5):424–32. 10.1111/j.1525-142X.2006.00116.x 16925678

[29] Ramsey M, Shoemaker C, Crews D. Gonadal expression of *Sf1* and *Aromatase* during sex determination in the red-eared slider turtle (*Trachemys scripta*), a reptile with temperature-dependent sex determination. Differentiation. 2007;75:978–91. 10.1111/j.1432-0436.2007.00182.x 17490415

[30] Torres Maldonado LC, Landa Piedra A, Moreno Mendoza N, Marmolejo Valencia A, Meza Martinez A, Merchant Larios H. Expression profiles of *Dax1*, *Dmrt1*, and *Sox9* during temperature sex determination in gonads of the sea turtle *Lepidochelys olivacea*. Gen Comp Endocrinol. 2003;129:20–6. 10.1016/s0016-6480(02)00511-7 12409092

[31] Valenzuela N. Evolution of the gene network underlying gonadogenesis in turtles with temperature-dependent and genotypic sex determination. Integr Comp Biol. 2008;48(4):476–85. 10.1093/icb/icn031 21669808

[32] Barske LA, Capel B. Estrogen represses SOX9 during sex determination in the red-eared slider turtle Trachemys scripta. Dev Biol. 2010;341(1):305–14. 10.1016/j.ydbio.2010.02.010 20153744

[33] Matsumoto Y, Yatsu R, Taylor C, Crews D. Changes in gonadal gene network by exogenous ligands in temperature-dependent sex determination. J Mol Endocrinol. 2013;50(3):389–400. 10.1530/JME-12-0260 23532621

[34] Valenzuela N. Multivariate expression analysis of the gene network underlying sexual development in turtle embryos with temperature-dependent and genotypic sex determination Sex Dev. 2010;4(1–2):39–49. 10.1159/000277935 20110645

[35] Valenzuela N, Shikano T. Embryological ontogeny of Aromatase gene expression in *Chrysemys picta* and *Apalone mutica* turtles: comparative patterns within and across temperature-dependent and genotypic sex-determining mechanisms. Dev Genes Evol. 2007;217:55–62. 10.1007/s00427-006-0106-3 17021865

[36] Matsumoto Y, Buemio A, Chu R, Vafaee M, Crews D. Epigenetic control of gonadal aromatase *cyp19a1* in temperature-dependent sex determination of red-eared slider turtles. PLoS ONE. 2013;8(6):e63599 10.1371/journal.pone.0063599 23762231 PMC3676416

[37] Kettlewell JR, Raymond CS, Zarkower D. Temperature-dependent expression of turtle *Dmrt1* prior to sexual differentiation. Genesis. 2000;26:174–8. 10705377

[38] Bergeron JM, Gahr M, Horan K, Wibbels T, Crews D. Cloning and in situ hybridization analysis of *estrogen receptor* in the developing gonad of the red-eared slider turtle, a species with temperature-dependent sex determination. Dev Growth Differ. 1998;40(2):243–54. 9572366 10.1046/j.1440-169x.1998.00013.x

[39] Chávez B, Ramos L, Merchant-Larios H, Vilchis F. Cloning and expression of the estrogen receptor-alpha (*Esr1*) from the Harderian gland of the sea turtle (*Lepidochelys olivacea*). Gen Comp Endocrinol. 2009;162:203–9. 10.1016/j.ygcen.2009.02.010 19272391

[40] Schroeder AL, Metzger KJ, Miller A, Rhen T. A novel candidate gene for temperature-dependent sex determination in the common snapping turtle. Genetics. 2016:115 182840. 10.1534/genetics.115.182840 PMC4858799 26936926

[41] Rhen T, Metzger K, Schroeder A, Woodward R. Expression of putative sex-determining genes during the thermosensitive period of gonad development in the snapping turtle, *Chelydra serpentina*. Sex Dev. 2007;1(4):255–70. 10.1159/000104775 18391536

[42] Valenzuela N, Neuwald JL, Literman R. Transcriptional evolution underlying vertebrate sexual development. Dev Dyn. 2013;242(4):307–19. 10.1002/dvdy.23897 23108853

[43] Cristino AS, Tanaka ED, Rubio M, Piulachs MD, Belles X. Deep sequencing of organ- and stage-specific micrornas in the evolutionarily basal insect *Blattella germanica* (l.) (Dictyoptera, Blattellidae). PLoS ONE. 2011;6(4). 10.1371/journal.pone.0019350 PMC3084283 21552535

[44] Devonshire AS, Sanders R, Wilkes TM, Taylor MS, Foy CA, Huggett JF. Application of next generation qPCR and sequencing platforms to mRNA biomarker analysis. Methods. 2013;59(1):89–100. 10.1016/j.ymeth.2012.07.021 22841564

[45] Valenzuela N, Badenhorst D, Montiel Jiménez EE, Literman R. Molecular cytogenetic search for cryptic sex chromosomes in painted turtles *Chrysemys picta*. Cytogenet Genome Res. 2014;144:39–46. 10.1159/000366076 25170556

[46] Bull JJ, Vogt RC. Temperature-sensitive periods of sex determination in emydid turtles. J Exp Zool. 1981;218(3):435–40. 10.1002/jez.1402180315 7338727

[47] Valenzuela N, Janzen FJ. Nest-site philopatry and the evolution of temperature-dependent sex determination. Evol Ecol Res. 2001;3:779–94.

[48] Milne-Morjan C, Valenzuela N. Is ground-nuzzling by female turtles associated with soil surface temperatures? J Herpetol. 2001;35(4):668–72.

[49] Morjan CL. Variation in nesting patterns affecting nest temperatures in two populations of painted turtles (*Chrysemys picta*) with temperature-dependent sex determination. Behav Ecol Sociobiol. 2003;53(4):254–61.

[50] Valenzuela N. The painted turtle, *Chrysemys picta*: A model system for vertebrate evolution, ecology, and human health. Cold Spring Harbor Protocols. 2009;4(7):1–9. 10.1101/pdb.emo124 20147199

[51] Shaffer HB, Minx P, Warren DE, Shedlock AM, Thomson RC, Valenzuela N, et al The western painted turtle genome, a model for the evolution of extreme physiological adaptations in a slowly evolving lineage. Genome Biol. 2013;14(3):R28: 1–2. 10.1186/gb-2013-14-3-r28 23537068 PMC4054807

[52] Badenhorst D, Hillier LW, Literman R, Montiel EE, Radhakrishnan S, Shen Y, et al Physical mapping and refinement of the painted turtle genome (*Chrysemys picta*) inform amniote genome evolution and challenge turtle-bird chromosomal conservation. Genome Biol Evol. 2015;7(7):2038–50. 10.1093/gbe/evv119 26108489 PMC4524486

[53] Shedlock AM, Botka CW, Zhao SY, Shetty J, Zhang TT, Liu JS, et al Phylogenomics of nonavian reptiles and the structure of the ancestral amniote genorne. Proc Natl Acad Sci U S A. 2007;104(8):2767–72. 10.1073/pnas.0606204104 17307883 PMC1815256

[54] Badenhorst D, Stanyon R, Engstrom T, Valenzuela N. A ZZ/ZW microchromosome system in the spiny softshell turtle, *Apalone spinifera*, reveals an intriguing sex chromosome conservation in Trionychidae. Chromosome Res. 2013;21(2):137–47. 10.1007/s10577-013-9343-2 23512312

[55] Bull JJ, Vogt RC. Temperature-dependent sex determination in turtles. Science. 1979;206:1186–8. 505003 10.1126/science.505003

[56] Morrish BC, Sinclair AH. Vertebrate sex determination: many means to an end. Reproduction. 2002;124:447–57. 12361462 10.1530/rep.0.1240447

[57] Kuroki S, Matoba S, Akiyoshi M, Matsumura Y, Miyachi H, Mise N, et al Epigenetic regulation of mouse sex determination by the histone demethylase *Jmjd1a*. Science. 2013;341(6150):1106–9. 10.1126/science.1239864 24009392

[58] Carmi I, Kopczynski JB, Meyer BJ. The nuclear hormone receptor *Sex-1* is an X-chromosome signal that determines nematode sex. Nature. 1998;396:168–73. 10.1038/24164 9823896

[59] Kohno S, Katsu Y, Urushitani H, Ohta Y, Iguchi T, Guillette LJ. Potential contributions of heat shock proteins to temperature-dependent sex determination in the american alligator. Sex Dev. 2010;4(1–2):73–87. 10.1159/000260374 19940440 PMC2855287

[60] Vogt RC, Bull JJ. Genetic sex determination in the spiny softshell *Trionyx spiniferus* (Testudines, Trionychidae). Copeia. 1982;(3):699–700.

[61] Valenzuela N. Egg incubation and collection of painted turtle embryos. Cold Spring Harbor Protocols. 2009;4(7):1–3. 10.1101/pdb.prot5238 20147203

[62] Liu C, Rodriguez K, Yao HH-C. Mapping lineage progression of somatic progenitor cells in the mouse fetal testis. Development. 2016;143(20):3700–10. 10.1242/dev.135756 27621062 PMC5087644

[63] Merchant-Larios H, Diaz-Hernandez V, Marmolejo-Valencia A. Gonadal morphogenesis and gene expression in reptiles with temperature-dependent sex determination. Sex Dev. 2010;4(1–2):50–61. 10.1159/000276768 20090307

[64] Valenzuela N. Constant, shift and natural temperature effects on sex determination in *Podocnemis expansa* turtles. Ecology. 2001;82(11):3010–24.

[65] Gómez-Saldarriaga C, Valenzuela N, Ceballos CP. Effects of incubation temperature on sex determination in the endangered magdalena river turtle, *Podocnemis lewyana*. Chelonian Conserv Biol. 2016;15(1):43–53.

[66] Wu TD, Nacu S. Fast and SNP-tolerant detection of complex variants and splicing in short reads. Bioinformatics. 2010;26(7):873–81. 10.1093/bioinformatics/btq057 20147302 PMC2844994

[67] Bolger AM, Lohse M, Usadel B. Trimmomatic: a flexible trimmer for Illumina sequence data. Bioinformatics. 2014;30(15):2114–20. 10.1093/bioinformatics/btu170 24695404 PMC4103590

[68] Grabherr MG, Haas BJ, Yassour M, Levin JZ, Thompson DA, Amit I, et al Full-length transcriptome assembly from RNA-Seq data without a reference genome. Nat Biotechnol. 2011;29(7):644–U130. 10.1038/nbt.1883 21572440 PMC3571712

[69] Haas BJ, Papanicolaou A, Yassour M, Grabherr M, Blood PD, Bowden J, et al De novo transcript sequence reconstruction from RNA-seq using the Trinity platform for reference generation and analysis. Nature Protocols. 2013;8(8):1494–512. 10.1038/nprot.2013.084 23845962 PMC3875132

[70] Boeckmann B, Bairoch A, Apweiler R, Blatter MC, Estreicher A, Gasteiger E, et al The SWISS-PROT protein knowledgebase and its supplement TrEMBL in 2003. Nucleic Acids Res. 2003;31(1):365–70. 12520024 10.1093/nar/gkg095 PMC165542

[71] Wu TD, Watanabe CK. GMAP: a genomic mapping and alignment program for mRNA and EST sequences. Bioinformatics. 2005;21(9):1859–75. 10.1093/bioinformatics/bti310 15728110

[72] Kaplinsky NJ, Gilbert SF, Cebra-Thomas J, Lillevali K, Saare M, Chang EY, et al The embryonic transcriptome of the red-eared slider turtle (*Trachemys scripta*). PLoS ONE. 2013;8. 10.1371/journal.pone.0066357 PMC3686863 23840449

[73] Anders S, Pyl PT, Huber W. HTSeq A Python framework to work with high-throughput sequencing data. bioRxiv preprint. 2014:0–5. 10.1093/bioinformatics/btu638 PMC4287950 25260700

[74] R Core Development Team. R: a language and environment for statistical computing. Version 3.2.2 ed. Vienna: R Foundation for Statistical Computing. Available via http://cran.R-project.org.; 2012.

[75] Bullard JH, Purdom E, Hansen KD, Dudoit S. Evaluation of statistical methods for normalization and differential expression in mRNA-Seq experiments. BMC Bioinformatics. 2010;11:94-. 10.1186/1471-2105-11-94 20167110 PMC2838869

[76] Auer PL, Doerge RW. Statistical design and analysis of RNA sequencing data. Genetics. 2010;185(2):405–U32. 10.1534/genetics.110.114983 20439781 PMC2881125

[77] Al Seesi S, Tiagueu YT, Zelikovsky A, Mandoiu II. Bootstrap-based differential gene expression analysis for RNA-Seq data with and without replicates. BMC Genomics. 2014;15. 10.1186/1471-2164-15-S8-S2 PMC4248812 25435284

[78] Benjamini Y, Hochberg Y. Controlling the false discovery rate—A practical and powerful approach to multiple testing. Journal of the Royal Statistical Society Series B-Methodological. 1995;57(1):289–300.

[79] Kanehisa M. The KEGG database. Novartis Found Symp. 2002;247:91–101; discussion -3, 19–28, 244–52. 12539951

[80] Huang DW, Sherman BT, Tan Q, Kir J, Liu D, Bryant D, et al DAVID Bioinformatics Resources: expanded annotation database and novel algorithms to better extract biology from large gene lists. Nucleic Acids Res. 2007;35:W169–W75. 10.1093/nar/gkm415 17576678 PMC1933169

[81] Liu Y, Zhou J, White KP. RNA-seq differential expression studies: more sequence or more replication? Bioinformatics. 2014;30(3):301–4. 10.1093/bioinformatics/btt688 24319002 PMC3904521

[82] Anders S. Analysing RNA-Seq data with the DESeq package. R manual. 2012:1–28.

[83] Robinson MD, McCarthy DJ, Smyth GK. edgeR: a Bioconductor package for differential expression analysis of digital gene expression data. Bioinformatics. 2010;26(1):139–40. 10.1093/bioinformatics/btp616 19910308 PMC2796818

[84] Langfelder P, Horvath S. WGCNA: an R package for weighted correlation network analysis. BMC Bioinformatics. 2008;9:559–. 10.1186/1471-2105-9-559 19114008 PMC2631488

[85] Wang Z, Pascual-Anaya J, Zadissa A, Li W, Niimura Y, Huang Z, et al The draft genomes of soft-shell turtle and green sea turtle yield insights into the development and evolution of the turtle-specific body plan. Nat Genet. 2013;45(6):701–6. 10.1038/ng.2615 23624526 PMC4000948

[86] Wang C, Tang X, Xin Y, Yue F, Yan X, Liu B, et al Identification of sex chromosomes by means of comparative genomic hybridization in a lizard, *Eremias multiocellata*. Zool Sci. 2015;32(2):151–6. 10.2108/zs140246 25826063

[87] Szymanski M, Erdmann VA, Barciszewski J. Noncoding regulatory RNAs database. Nucleic Acids Res. 2003;31(1):429–31. 12520042 10.1093/nar/gkg124 PMC165571

[88] Camacho C, Coulouris G, Avagyan V, Ma N, Papadopoulos J, Bealer K, et al BLAST+: architecture and applications. BMC Bioinformatics. 2009;10:421–. 10.1186/1471-2105-10-421 20003500 PMC2803857

[89] Eggers S, Ohnesorg T, Sinclair A. Genetic regulation of mammalian gonad development. Nat Rev Endocrinol. 2014;10(11):673–83. 10.1038/nrendo.2014.163 25246082

[90] Robinson MD, Smyth GK. Moderated statistical tests for assessing differences in tag abundance. Bioinformatics. 2007;23(21):2881–7. 10.1093/bioinformatics/btm453 17881408

[91] Keenan SW, Hill CA, Kandoth C, Buck LT, Warren DE. Transcriptomic Responses of the Heart and Brain to Anoxia in the Western Painted Turtle. PLoS ONE. 2015;10(7):e0131669 10.1371/journal.pone.0131669 26147940 PMC4493013

[92] Girondot M, Delmas V, Rivalan P, Courchamp F, Prévot-Julliard AC, Godfrey MH. Implications of temperature-dependent sex determination for population dynamics In: Valenzuela N, Lance VA, editors. Temperature dependent sex determination in vertebrates. Washington, DC.: Smithsonian Books; 2004 p. 148–55.

[93] Literman R, Badenhorst D, Valenzuela N. qPCR-based molecular sexing by copy number variation in rRNA genes and its utility for sex identification in soft-shell turtles. Metod Ecol Evol. 2014;5(9):872–80.

[94] Ramsey M, Crews D. Steroid signaling and temperature-dependent sex determination—Reviewing the evidence for early action of estrogen during ovarian determination in turtles. Semin Cell Dev Biol. 2009;20(3):283–92. 10.1016/j.semcdb.2008.10.004 18992835 PMC2695493

[95] Smith CA, Shoemaker CM, Roeszler KN, Queen J, Crews D, Sinclair AH. Cloning and expression of R-Spondin1 in different vertebrates suggests a conserved role in ovarian development. BMC Dev Biol. 2008;8:72 10.1186/1471-213X-8-72 18651984 PMC2519078

[96] Bachvarova RF, Crother BI, Manova K, Chatfield J, Shoemaker CM, Crews DP, et al Expression of Dazl and Vasa in turtle embryos and ovaries: evidence for inductive specification of germ cells. Evol Dev. 2009;11(5):525–34. 10.1111/j.1525-142X.2009.00360.x 19754709

[97] Shoemaker-Daly CM, Jackson K, Yatsu R, Matsumoto Y, Crews D. Genetic Network Underlying Temperature-Dependent Sex Determination Is Endogenously Regulated by Temperature in Isolated Cultured Trachemys scripta Gonads. Dev Dyn. 2010;239(4):1061–75. 10.1002/dvdy.22266 20235200

[98] Weiner SA, Galbraith DA, Adams DC, Valenzuela N, Noll FB, Grozinger CM, et al A survey of DNA methylation across social insect species, life stages, and castes reveals abundant and caste-associated methylation in a primitively social wasp. Naturwissenschaften. 2013;100(8):795–9. 10.1007/s00114-013-1064-z 23793297

[99] Bogani D, Siggers P, Brixey R, Warr N, Beddow S, Edwards J, et al Loss of mitogen-activated protein kinase kinase kinase 4 (*Map3k4*) reveals a requirement for MAPK signalling in mouse sex determination. PLoS Biol. 2009;7. 10.1371/journal.pbio.1000196 PMC2733150 19753101

[100] Warr N, Carre GA, Siggers P, Faleato JV, Brixey R, Pope M, et al *Gadd45γ* and *Map3k4* interactions regulate mouse testis determination via p38 mapk-mediated control of *Sry* expression. Dev Cell. 2012;23:1020–31. 10.1016/j.devcel.2012.09.016 23102580 PMC3526779

[101] Kawagoshi T, Uno Y, Matsubara K, Matsuda Y, Nishida C. The ZW micro-sex chromosomes of the chinese soft-shelled turtle (*Pelodiscus sinensis*, Trionychidae, Testudines) have the same origin as chicken chromosome 15. Cytogenet Genome Res. 2009;125(2):125–31. 10.1159/000227837 19729916

[102] Chocu S, Calvel P, Rolland AD, Pineau C. Spermatogenesis in mammals: proteomic insights. Sys Biol Rep Med. 2012;58:179–90. 10.3109/19396368.2012.691943 22788530

[103] Pires-daSilva A, Sommer RJ. The evolution of signalling pathways in animal development. Nat Rev Genet. 2003;4(1):39–49. 10.1038/nrg977 12509752

[104] Hou SX, Zheng Z, Chen X, Perrimon N. The JAK/STAT pathway in model organisms: Emerging roles in cell movement. 2002. p. 765–78. 10.1016/s1534-5807(02)00376-3 12479803

[105] Hayden MS, West AP, Ghosh S. *Nf-kappaB* and the immune response. Oncogene. 2006;25(51):6758–80. 10.1038/sj.onc.1209943 17072327

[106] Chandel NS, Trzyna WC, McClintock DS, Schumacker PT. Role of oxidants in *Nf-kappa B* activation and *Tnf-alpha* gene transcription induced by hypoxia and endotoxin. J Immunol. 2000;165(2):1013–21. 10878378 10.4049/jimmunol.165.2.1013

[107] Korus M, Mahon GM, Cheng L, Whitehead IP. *p38 MAPK*-mediated activation of *Nf-kappaB* by the *RhoGEF* domain of Bcr. Oncogene. 2002;21(30):4601–12. 10.1038/sj.onc.1205678 12096337

[108] Bott RC, Clopton DT, Cupp AS. A proposed role for *Vegf* isoforms in sex-specific vasculature development in the gonad. Reprod Dom Anim. 2008;43(SUPPL.2):310–6. 10.1111/j.1439-0531.2008.01179.x PMC2708602 18638140

[109] Mu X, Wen J, Guo M, Wang J, Li G, Wang Z, et al *Retinoic acid* derived from the fetal ovary initiates meiosis in mouse germ cells. J Cell Physiol. 2013;228(3):627–39. 10.1002/jcp.24172 22886539

[110] Ricken A, Lochhead P, Kontogiannea M, Farookhi R. *Wnt* signaling in the ovary: Identification and compartmentalized expression of *Wnt-2*, *Wnt-2b*, and *Frizzled-4* mRNAs. Endocrinology. 2002;143:2741–9. 10.1210/endo.143.7.8908 12072409

[111] Nef S, Vassalli J-D. Complementary pathways in mammalian female sex determination. J Biol. 2009;8:74–. 10.1186/jbiol173 19735582 PMC2776915

[112] Bernard P, Ryan J, Sim H, Czech DP, Sinclair AH, Koopman P, et al *Wnt* signaling in ovarian development inhibits *Sf1* activation of Sox9 via the *Tesco* enhancer. Endocrinology. 2012;153:901–12. 10.1210/en.2011-1347 22128028

[113] Sundaram M, Yochem J, Han M. A *Ras*-mediated signal transduction pathway is involved in the control of sex myoblast migration in *Caenorhabditis elegans*. Development. 1996;122:2823–33. 8787756 10.1242/dev.122.9.2823

[114] Roos-Mattjus P, Sistonen L. The *ubiquitin*-proteasome pathway. Ann Med. 2004;36:285–95. 15224655 10.1080/07853890310016324

[115] Liu CF, Bingham N, Parker K, Yao HHC. Sex-specific roles of *beta-catenin* in mouse gonadal development. Hum Mol Genet. 2009;18(3):405–17. 10.1093/hmg/ddn362 18981061 PMC2638797

[116] Chassot AA, Bradford ST, Auguste A, Gregoire EP, Pailhoux E, de Rooij DG, et al. Wnt4 and Rspo1 together are required for cell proliferation in the early mouse gonad. 2012. 10.1242/dev.078972 23095882

[117] Nef S, Verma-Kurvari S, Merenmies J, Vassalli JD, Efstratiadis A, Accili D, et al Testis determination requires *insulin receptor* family function in mice. Nature. 2003;426(6964):291–5. 10.1038/nature02059 14628051

[118] Mittwoch U. The elusive action of sex-determining genes: mitochondria to the rescue? J Theor Biol. 2004;228(3):359–65. 10.1016/j.jtbi.2004.02.001 15135034

[119] Charnov EL, Bull JJ. When is sex environmentally determined? Nature. 1977;266:828–30. 865602 10.1038/266828a0

[120] Hedges SB, Marin J, Suleski M, Paymer M, Kumar S. Tree of Life Reveals Clock-Like Speciation and Diversification. Mol Biol Evol. 2015;32(4):835–45. 10.1093/molbev/msv037 25739733 PMC4379413

[121] Bieser KL, Wibbels T. Chronology, magnitude and duration of expression of putative sex-determining/differentiation genes in a turtle with temperature-dependent sex determination. Sex Dev. 2014;8(6):364–75. 10.1159/000369116 25427533

[122] Kamiya T, Kai W, Tasumi S, Oka A, Matsunaga T, Mizuno N, et al A trans-species missense SNP in Amhr2 is associated with sex determination in the tiger pufferfish, *Takifugu rubripes* (Fugu). PLoS Genet. 2012;8(7). 10.1371/journal.pgen.1002798 PMC3395601 22807687

[123] Yamamoto Y, Zhang Y, Sarida M, Hattori RS, Struessmann CA. Coexistence of genotypic and temperature-dependent sex determination in pejerrey *Odontesthes bonariensis*. PLoS ONE. 2014;9(7). 10.1371/journal.pone.0102574 PMC4103838 25036903

[124] Graves JAM, Peichel CL. Are homologies in vertebrate sex determination due to shared ancestry or to limited options? Genome Biol. 2010;11(4). 10.1186/gb-2010-11-4-205 PMC2884537 20441602

[125] Minkina A, Matson CK, Lindeman RE, Ghyselinck NB, Bardwell VJ, Zarkower D. DMRT1 protects male gonadal cells from retinoid-dependent sexual transdifferentiation. Dev Cell. 2014;29(5):511–20. 10.1016/j.devcel.2014.04.017 24856513 PMC4105363

[126] Janes DE, Organ CL, Stiglec R, O'Meally D, Sarre SD, Georges A, et al Molecular evolution of *Dmrt1* accompanies change of sex-determining mechanisms in reptilia. Biol Lett. 2014;10(12):20140809-. 10.1098/rsbl.2014.0809 25540158 PMC4298190

[127] Fish EN. The X-files in immunity: sex-based differences predispose immune responses. Nature reviews Immunology. 2008;8(9):737–44. 10.1038/nri2394 18728636 PMC7097214

[128] Mittwoch U. Sex determination. EMBO Rep. 2013;14(7):588–92. 10.1038/embor.2013.84 23764925 PMC3701250

[129] Pelletier J, Schalling M, Buckler AJ, Rogers A, Haber DA, Housman D. Expression of the Wilms' tumor gene Wt1 in the murine urogenital system. Genes and Development. 1991;5(8):1345–56. 1651275 10.1101/gad.5.8.1345

[130] Oreal E, Mazaud S, Picard JY, Magre S, Carre-Eusebe D. Different patterns of *anti-Mullerian hormone* expression, as related to *Dmrt1*, *Sf-1*, *Wt1*, *Gata-4*, *Wnt-4*, and *Lhx9* expression, in the chick differentiating gonads. Dev Dyn. 2002;225(3):221–32. 10.1002/dvdy.10153 12412004

[131] Pieau C, Dorizzi M. Oestrogens and temperature-dependent sex determination in reptiles: all is in the gonads. J Endocrinol. 2004;181(3):367–77. 15171684 10.1677/joe.0.1810367

[132] Ramsey M, Crews D. Adrenal-kidney-gonad complex measurements may not predict gonad-specific changes in gene expression patterns during temperature-dependent sex determination in the red-eared slider turtle (Trachemys scripta elegans). J Exp Zool Part A Ecol Genet Physiol. 2007;307A(8):463–70. 10.1002/jez.399 17592622

[133] Shoemaker C, Ramsey M, Queen J, Crews D. Expression of *Sox9*, *Mis*, and *Dmrt1* in the gonad of a species with temperature-dependent sex determination. Dev Dyn. 2007;236:1055–63. 10.1002/dvdy.21096 17326219

[134] Parker KL, Schimmer BP. *Steroidogenic factor 1*: A key determinant of endocrine development and function. Endocr Rev. 1997;18(3):361–77. 10.1210/edrv.18.3.0301 9183568

[135] Crews D, Fleming A, Willingham E, Baldwin R, Skipper JK. Role of *steroidogenic factor 1* and *aromatase* in temperature- dependent sex determination in the red-eared slider turtle. J Exp Zool. 2001;290(6):597–606. 11748608 10.1002/jez.1110

[136] Ramkissoon Y, Goodfellow P. Early steps in mammalian sex determination. Curr Opin Genet Dev. 1996;6(3):316–21. 8791510 10.1016/s0959-437x(96)80008-6

[137] Meeks JJ, Weiss J, Jameson JL. Dax1 is required for testis determination. Nat Genet. 2003;34(1):32–3. 10.1038/ng1141 12679814

[138] Hughes IA, Coleman N, Ahmed SF, Ng KL, Cheng A, Lim HN, et al Sexual dimorphism in the neonatal gonad. Acta Paediatrica. 1999;88:23–30. 10.1111/j.1651-2227.1999.tb14347.x 10102048

[139] Castro LFC, Santos MM, Reis-Henriques MA. The genomic environment around the Aromatase gene: evolutionary insights. BMC Evol Biol. 2005;5. 10.1186/1471-2148-5-43 PMC1215479 16098224

[140] George FW, Wilson JD. Conversion of androgen to estrogen by human fetal ovary. Journal of Clinical Endocrinology & Metabolism. 1978;47(3):550–5. 263310 10.1210/jcem-47-3-550

[141] Smith CA, Sinclair AH. Sex determination in the chicken embryo. J Exp Zool. 2001;290:691–9. 11748617 10.1002/jez.1119

[142] Nanda I, Haaf T, Schartl M, Schmid M, Burt DW. Comparative mapping of Z-orthologous genes in vertebrates: implications for the evolution of avian sex chromosomes. Cytogenet Genome Res. 2002;99(1–4):178–84. 12900562 10.1159/000071591

[143] Smith CA, Roeszler KN, Ohnesorg T, Cummins DM, Farlie PG, Doran TJ, et al The avian Z-linked gene DMRT1 is required for male sex determination in the chicken. Nature (Lond). 2009;461(7261):267–71. 19710650 10.1038/nature08298

[144] Miqueloto CA, Zorn TMT. Characterization and distribution of hyaluronan and the proteoglycans decorin, biglycan and perlecan in the developing embryonic mouse gonad. J Anat. 2007;211(1):16–25. 10.1111/j.1469-7580.2007.00741.x 17543016 PMC2375803

[145] Washietl S, Hofacker IL, Lukasser M, Huttenhofer A, Stadler PF. Mapping of conserved RNA secondary structures predicts thousands of functional noncoding RNAs in the human genome. Nat Biotech. 2005;23(11):1383–90. 10.1038/nbt1144 16273071

[146] Disteche CM. Dosage Compensation of the Sex Chromosomes. Annu Rev Genet. 2012;46:537–60. 10.1146/annurev-genet-110711-155454 22974302 PMC3767307

[147] Costanzo JP, Jones EE, Lee RE. Physiological responses to supercooling and hypoxia in the hatchling painted turtle, Chrysemys picta. J Comp Physiol B Biochem Syst Environ Physiol. 2001;171(4):335–40. 10.1007/s003600100181 11409631

[148] Palmieri F. The mitochondrial transporter family SLC25: Identification, properties and physiopathology. 2013. p. 465–84. 10.1016/j.mam.2012.05.005 23266187

[149] Zhang W-H, Wang X, Narayanan M, Zhang Y, Huo C, Reed JC, et al Fundamental role of the *Rip2/caspase-1* pathway in hypoxia and ischemia-induced neuronal cell death. Proc Natl Acad Sci U S A. 2003;100(26):16012–7. 10.1073/pnas.2534856100 14663141 PMC307684

[150] Zhou J, Damdimopoulos AE, Spyrou G, Brüne B. *Thioredoxin 1* and *Thioredoxin 2* have opposed regulatory functions on *Hypoxia-inducible factor-1α*. J Biol Chem. 2007;282(10):7482–90. 10.1074/jbc.M608289200 17220299

