# Supplementary material for: Transcriptomic responses to environmental temperature by turtles with temperature-dependent and genotypic sex determination assessed by RNAseq inform the genetic architecture of embryonic gonadal development
Source: PLoS One. 2017 Mar 15;12(3):e0172044. doi: 10.1371/journal.pone.0172044 (PMC5352168; doi:10.1371/journal.pone.0172044)

SUPPLEMENTAL FIGURE 1

Title: Transcriptomic responses to environmental temperature by turtles with thermosensitive and genotypic sex determination assessed by RNAseq inform the genetic architecture of embryonic gonadal development

Authors: Srihari Radhakrishnan[1, 4], Robert Literman[2, 4], Jennifer Neuwald[4, 5], Andrew Severin[3, 4], Nicole Valenzuela*[4]
***corresponding author; Email: nvalenzu@iastate.edu**

Figure S1: *Chrysemys picta* - Novel transcripts that are highly differentially-expressed (while controlling false discoveries at 1e-10). Blue: upregulated at 26°C, red: upregulated at 31°C.

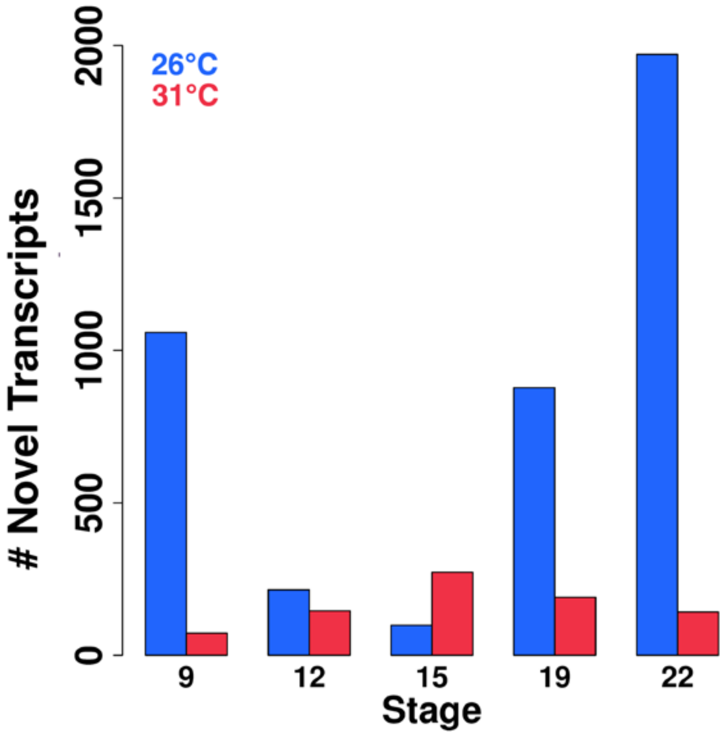

Supplement: S1 Fig — Blue: upregulated at 26°C, red: upregulated at 31°C. (PDF) [file pone.0172044.s001.pdf]
