# Supplementary material for: Transcriptomic responses to environmental temperature by turtles with temperature-dependent and genotypic sex determination assessed by RNAseq inform the genetic architecture of embryonic gonadal development
Source: PLoS One. 2017 Mar 15;12(3):e0172044. doi: 10.1371/journal.pone.0172044 (PMC5352168; doi:10.1371/journal.pone.0172044)

SUPPLEMENTAL FIGURE 2

Title: Transcriptomic responses to environmental temperature by turtles with thermosensitive and genotypic sex determination assessed by RNAseq inform the genetic architecture of embryonic gonadal development

Authors: Srihari Radhakrishnan[1, 4], Robert Literman[2, 4], Jennifer Neuwald[4, 5], Andrew Severin[3, 4], Nicole Valenzuela*[4]
***corresponding author; Email: nvalenzu@iastate.edu**

Figure S2: RNA-seq expression patterns for multiple genes previously implicated in mammalian urogonadal development across developmental stages 9-22 at 26°C (blue) and 31°C (red) in Chrysemys picta and Apalone spinifera. Statistically significant differences are indicated with an asterisk (*). Thermosensitive period is indicated by a box.

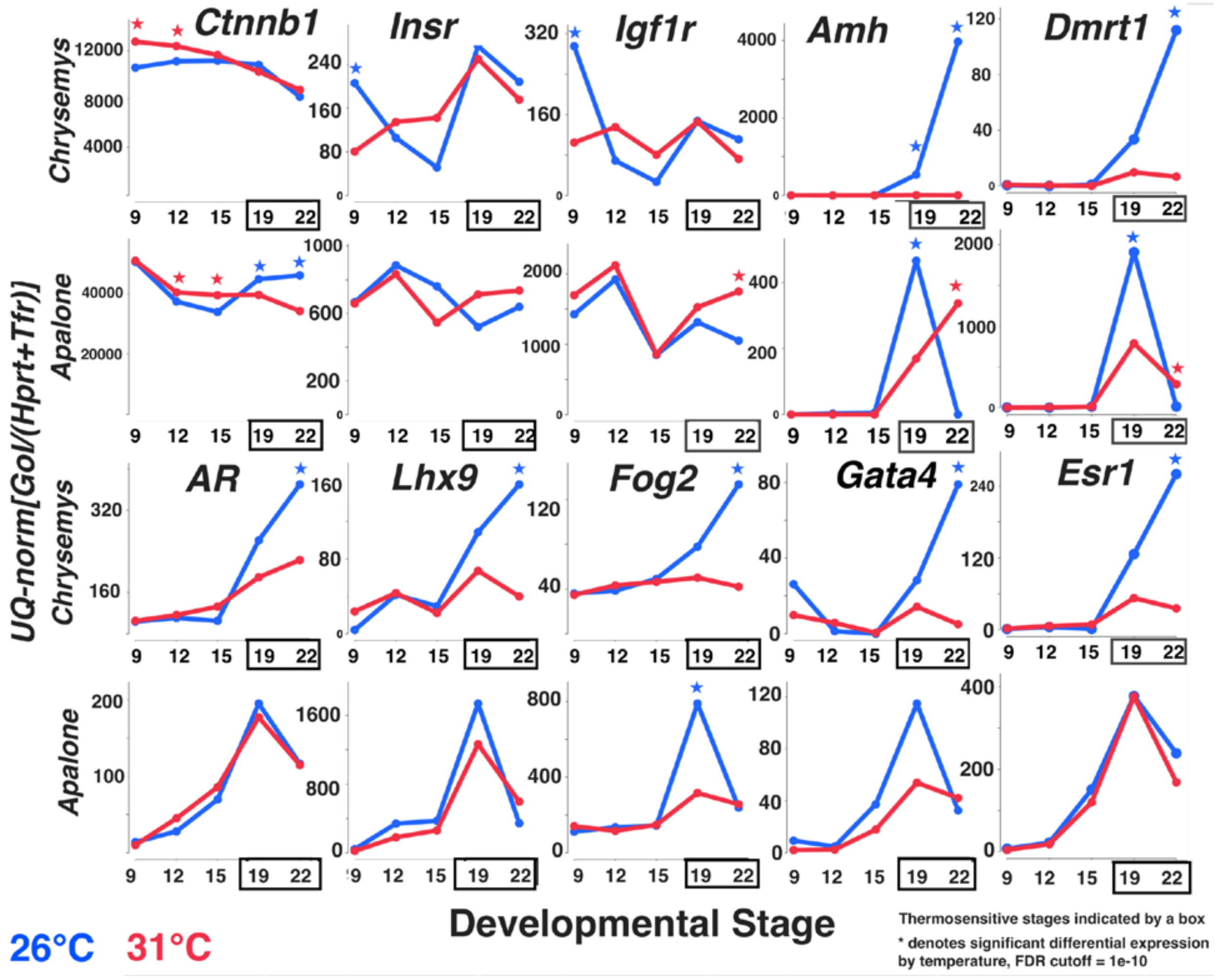

Supplement: S2 Fig — Statistically significant differences are indicated with an asterisk (*). Thermosensitive period is indicated by a box. (PDF) [file pone.0172044.s002.pdf]
