# Supplementary material for: Transcriptomic responses to environmental temperature by turtles with temperature-dependent and genotypic sex determination assessed by RNAseq inform the genetic architecture of embryonic gonadal development
Source: PLoS One. 2017 Mar 15;12(3):e0172044. doi: 10.1371/journal.pone.0172044 (PMC5352168; doi:10.1371/journal.pone.0172044)

SUPPLEMENTAL FIGURE 3

Title: Transcriptomic responses to environmental temperature by turtles with thermosensitive and genotypic sex determination assessed by RNAseq inform the genetic architecture of embryonic gonadal development

Authors: Srihari Radhakrishnan[1, 4], Robert Literman[2, 4], Jennifer Neuwald[4, 5], Andrew Severin[3, 4], Nicole Valenzuela*[4]
***corresponding author; Email: nvalenzu@iastate.edu**

Figure S3: Gene co-expression patterns by temperature for each turtle species from the RNA-seq data. Panels a-d illustrate modules of high (red) and low (yellow) co-expression for 981 genes of interest (described in Table 3) profiled across five developmental stages in *Chrysemys* picta [(a) and (b)] and *Apalone spinifera* [(c) and (dc)] at 26°C and 31°C respectively.

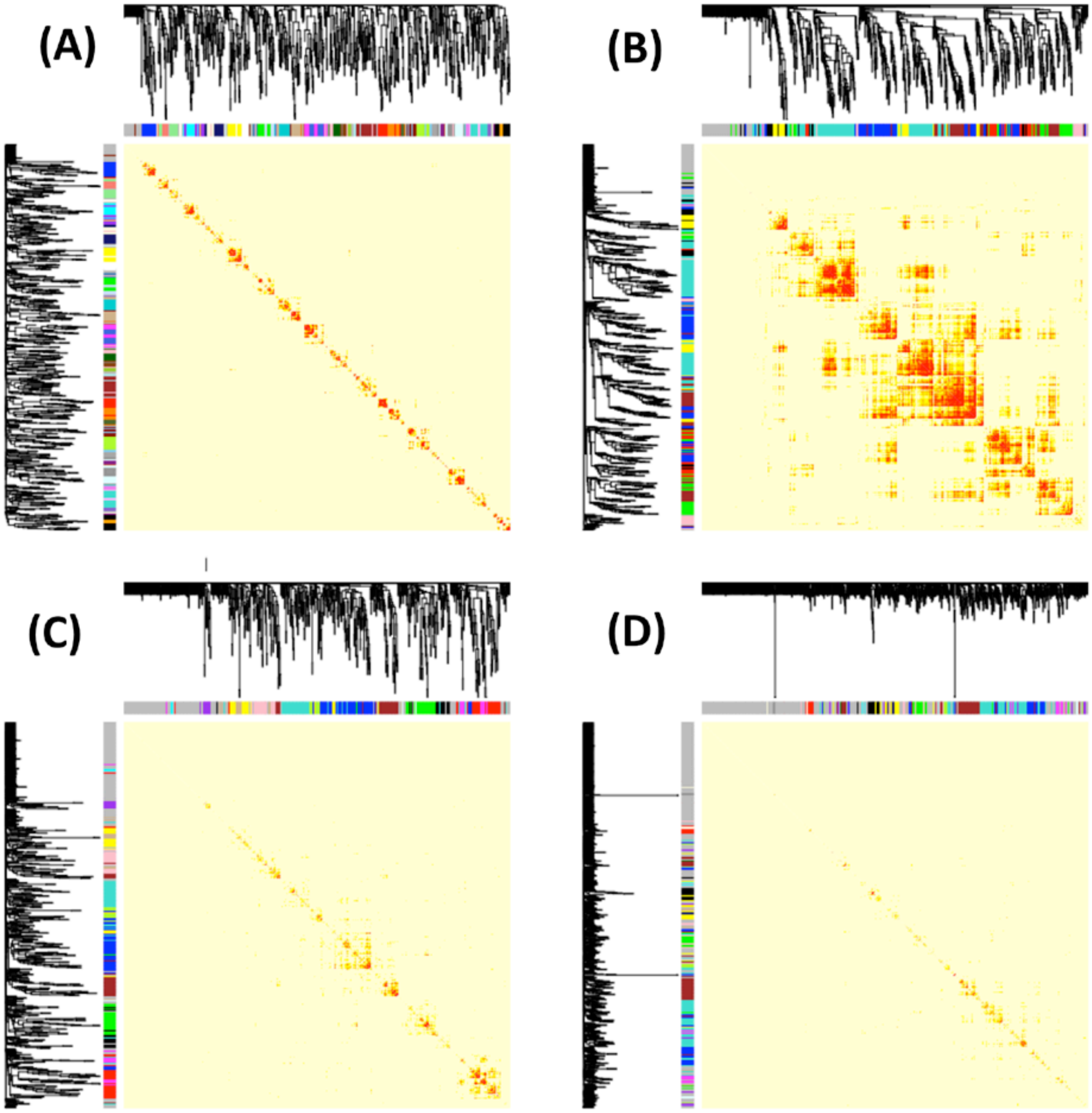

Supplement: S3 Fig — Panels a-d illustrate modules of high (red) and low (yellow) co-expression for 981 genes of interest (described in Table 5) profiled across five developmental stages in Chrysemys picta [(a) and (b)] and Apalone spinifera [(c) and (dc)] at 26°C and 31°C respectively. (PDF) [file pone.0172044.s003.pdf]
